# Cross‐modal integration of bulk RNA‐seq and single‐cell RNA sequencing data to reveal T‐cell exhaustion in colorectal cancer

**DOI:** 10.1111/jcmm.70101

**Published:** 2024-09-29

**Authors:** Mingcong Xu, Guorui Zhang, Ting Cui, Jiaqi Liu, Qiuyu Wang, Desi Shang, Tingting Yu, Bingzhou Guo, Jinjie Huang, Chunquan Li

**Affiliations:** ^1^ School of Computer Science and Technology Harbin University of Science and Technology Harbin China; ^2^ Cardiovascular Lab of Big Data and Imaging Artificial Intelligence, Hengyang Medical School, The First Affiliated Hospital, University of South China Hengyang Hunan China; ^3^ Insititute of Biochemistry and Molecular Biology, Hengyang Medical College, University of South China Hengyang Hunan China; ^4^ Hunan Provincial Key Laboratory of Multi‐Omics and Artificial Intelligence of Cardiovascular Diseases University of South China Hengyang Hunan China; ^5^ College of Artificial Intelligence and Big Data for Medical Sciences, Shandong First Medical University Jinan Shandong China

**Keywords:** bulk RNA‐seq, knowledge distillation, scRNA‐seq, T‐cell exhaustion

## Abstract

Colorectal cancer (CRC) is a relatively common malignancy clinically and the second leading cause of cancer‐related deaths. Recent studies have identified T‐cell exhaustion as playing a crucial role in the pathogenesis of CRC. A long‐standing challenge in the clinical management of CRC is to understand how T cells function during its progression and metastasis, and whether potential therapeutic targets for CRC treatment can be predicted through T cells. Here, we propose DeepTEX, a multi‐omics deep learning approach that integrates cross‐model data to investigate the heterogeneity of T‐cell exhaustion in CRC. DeepTEX uses a domain adaptation model to align the data distributions from two different modalities and applies a cross‐modal knowledge distillation model to predict the heterogeneity of T‐cell exhaustion across diverse patients, identifying key functional pathways and genes. DeepTEX offers valuable insights into the application of deep learning in multi‐omics, providing crucial data for exploring the stages of T‐cell exhaustion associated with CRC and relevant therapeutic targets.

## 1 INTRODUCTION

Colorectal cancer (CRC) is one of the most common malignancies worldwide.^1^ T‐cell exhaustion refers to a state of dysfunction in T cells, common in chronic infections and cancer patients.^2^ It is characterized by poor effector functions, persistent expression of inhibitory receptors and a transcriptional state distinct from functional effector or memory T cells.^3^ Research has found that T‐cell exhaustion typically undergoes four distinct stages.^4^, ^5^ The first stage is T‐cell exhaustion progenitors (TEXprog), where T cells are stationary in specific body locations and do not actively combat infections or tumours. The second and third stages are T‐cell exhaustion intermediates (TEXint1 and TEXint2), where T cells have lost some of their infection‐fighting ability but still possess some effector‐like characteristics. The fourth stage is T‐cell exhaustion terminally (TEXterm), where T cells become mature and specialized. In this stage, they are no longer able to replicate themselves or effectively combat viruses or tumours. Stage four is characterized by high levels of PD‐1 expression, indicating that T cells no longer respond to certain treatments. The ‘un‐exhausting’ of T cells in the tumour microenvironment (TME) is often considered a key mechanism of action for immune checkpoint inhibitors, whereas T‐cell exhaustion contributes to cell immunotherapy resistance.^6^

Single‐cell RNA sequencing (scRNA‐seq) technology allows the study of gene expression patterns at the single‐cell level, revealing cell heterogeneity within tissues.^7^ Compared to traditional bulk RNA‐seq, scRNA‐seq can identify and distinguish different cell populations more precisely, revealing their specific functions and states under disease conditions. Previous studies have provided scRNA‐seq data that can be used to analyse the state of T‐cell exhaustion within the TME.^8^, ^9^ Through scRNA‐seq, we can identify subpopulations of T cells expressing exhaustion‐related marker genes (such as PDCD1, TIGIT), as well as their spatial distribution and functional status in the TME.^10^ However, scRNA‐seq data itself may have sparsity and technical noise, which could affect the accuracy of data analysis.^11^ Accordingly, the incorporation of deep learning technology has emerged as a pivotal strategy for enhancing the quality of data analysis. Deep learning models are adept at recognizing complex data modalities and distilling meaningful information from biological big data.^12^, ^13^

In recent years, there has been a significant increase in research focused on the deconvolution algorithm, which aims to estimate cell heterogeneity and purity from bulk sequencing data.^14^, ^15^ Notable algorithms in this domain include CIBERSORT,^16^ xCell^17^ and ESTIMATE.^18^ However, there is a lack of effective machine learning methods specifically designed to predict the level of T‐cell exhaustion in CRC. Previous methods have primarily used gene expression profiles to infer cell heterogeneity, without considering the impact of pathways or functional genes.^19^

Here, we have developed DeepTEX, a novel deep learning modal that leverages domain adaptation and knowledge distillation to integrate scRNA‐seq data with bulk RNA‐seq data, which facilitates the identification of T‐cell exhaustion heterogeneity. By applying DeepTEX to bulk data from patients with CRC, the level of T‐cell exhaustion was successfully identified, providing a more comprehensive insight into the composition of the TME. Simultaneously, DeepTEX can identify cross‐modal functionally, significant pathways and functional gene sets, leveraging knowledge distillation and sparse regularization techniques. This offers potential biomarkers for clinical treatment and aids in developing more precise therapeutic strategies.

## 2 MATERIALS AND METHODS

### 2.1 Data source

To better explore the regulatory mechanisms of T‐cell exhaustion in primary CRC and metastatic cancer, we collected scRNA‐seq data associated with CRC patients from public databases. The cancer scRNA‐seq dataset GSE178318^20^ was downloaded from the Gene Expression Omnibus (GEO) database (https://www.ncbi.nlm.nih.gov/geo/); it includes sequencing sample data from primary, liver metastasis and blood‐derived CRC sources. After quality control, the scRNA‐seq samples included 6518 cells from primary CRC, 10,145 cells from CRC liver metastasis, and 2992 cells from peripheral blood metastasis. Extensive CRC transcriptome data and clinical information from The Cancer Genome Atlas (TCGA) database were downloaded from the GDC database (https://portal.gdc.cancer.gov/). For a relatively accurate assessment of factors associated with the survival of CRC patients, we screened the sample information. After removing samples that lacked survival information, 424 patients were selected for further analysis (Tables S1 and S2). We added the GSE159216^21^ validation data from the GEO database and ultimately retained 171 patient samples after quality control (detailed in Supplementary Methods 1.1).

### 2.2 DeepTEX model workflow for T‐cell exhaustion analysis

**FIGURE 1:**
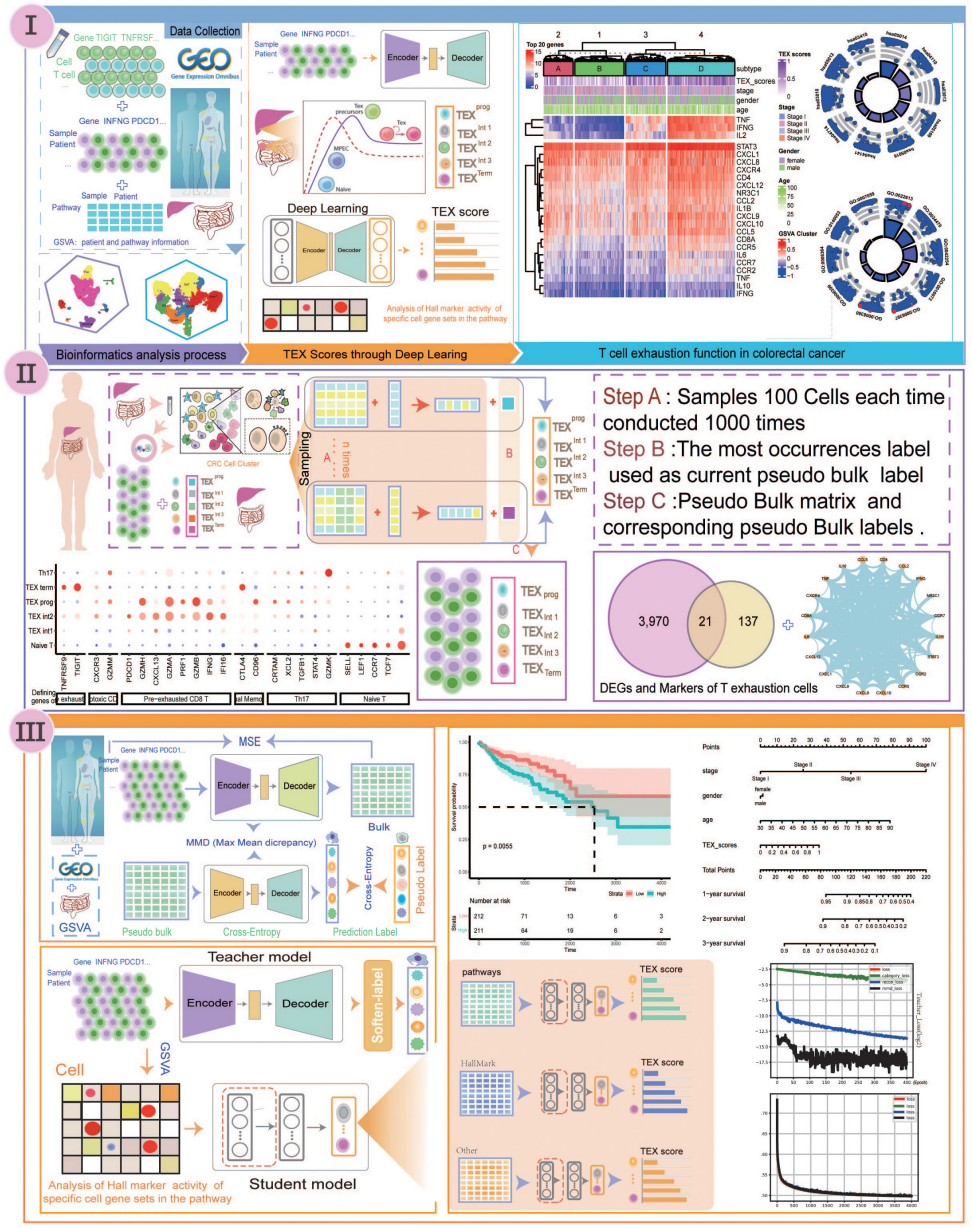
The complete workflow of DeepTEX.

Here, we introduce DeepTEX, a novel deep learning modal that integrates scRNA‐seq and batch data, aiming to identify the level of T‐cell depletion in CRC patients and detect the key genes or other functional gene sets involved (Figure 1).

#### 2.2.1 Step 1

Construction of pseudo‐bulk collections using scRNA‐seq data. The bulk data provides average gene expression information for the entire sample, whereas scRNA‐seq offers detailed insights at the single‐cell level. To simulate the distribution of bulk data, we randomly sample multiple blocks from the scRNA‐seq data. For each block, we average the gene expression levels of cells within it. Additionally, the most prevalent T‐cell exhaustion state in each block is selected as the label. Ultimately, a set of pseudo‐bulk samples, with rows representing genes and each sample accompanied by a pseudo‐label indicating the stage of T‐cell exhaustion are obtained.

#### 2.2.2 Step 2

Data are aligned using a domain adaptation model. Although pseudo‐bulk data were constructed using scRNA‐seq, there still exists a significant difference in the distribution between this pseudo‐bulk data and the actual bulk data. Therefore, we proposed a domain adaptation model to align the distributions of these two different types of data. The domain adaptation model consists of two parts: the source domain and the target domain. Its primary objective is to align the distributions of the source and target domains as closely as possible. To achieve this, it is essential to ensure that the feature dimensions of both domains are consistent. The use of multi‐layer neural networks to extract the same low‐dimensional feature dimensions has been effective. Therefore, we used an autoencoder (AE) to learn the distribution of the actual bulk data, which is composed of multiple layers of neural networks. We used a sigmoid activation function at the final output layer for predicting the original input. Concurrently, a category encoder (CE) is used to learn the distribution of the pseudo‐bulk data, which is also composed of multiple layers of neural networks. A softmax activation function is used at the final output layer to predict the proportions of different exhaustion stages. Finally, the most important issue is how to effectively align the low‐dimensional feature representations from different modalities. Here, we used the maximum mean discrepancy (MMD) loss to bring the distribution of the latent representations of the two datasets closer together.

#### 2.2.3 Step 3

The level of T‐cell exhaustion in CRC patients is predicted using a knowledge distillation model. For traditional deconvolution methods, such as CIBERSORT, a single‐modal gene expression is used for input features. However, for a complete biological process, the multiple genes of biological pathways are used. Therefore, converting gene expression profiles into pathway activity profiles can better reflect the importance of different biological processes. However, no method considers this well because both gene expression and pathway activity need to be addressed. How to train on two modalities and predict the level of T‐cell exhaustion on the pathway activity profile is a typical cross‐modal transfer problem. Knowledge distillation has been used to transfer different modalities and has achieved a good performance. It learns different modal representations from the teacher and student models and uses the knowledge learned by the teacher model to guide the student model. This can achieve good cross‐model knowledge transfer. Moreover, during the knowledge distillation process, the student model not only learns the characteristics of the original data but also acquires more general cross‐data feature representations from the teacher model, which helps to improve its ability to generalize. Therefore, we use the trained domain adaptation model as the teacher model and a new multi‐layer neural network as the student model. Meanwhile, we adopted boosting techniques to enhance the performance of the final model. The teacher model extracts feature representations by inputting actual bulk data into the AE model, and these extracted features are then used by the CE model to output soft labels (knowledge) for the level of T‐cell exhaustion. Subsequently, we converted bulk data into pathway activity matrices using GSVA,^22^ which serves as input for the student network to learn the knowledge extracted by the teacher model (Table S12). Our trained cross‐modal knowledge distillation model can predict the level of exhaustion in different CRC patients. Additionally, we added L1 regularization constraints to the first layer of the student model, aiming to infer related active pathways. Importantly, the input feature matrix for the student model can be various transformed activity matrices, such as pathway activity matrices, HALLMARK activity matrices, etc. Our model also supports the original gene expression matrix as input. Unlike the teacher network, which uses a filtered bulk matrix with selected genes, the student network input includes bulk matrices using all genes, allowing important genes related to T‐cell exhaustion to be identified. Here we have introduced the overall architecture of the model.

### 2.3 Single‐cell data preprocessing and multi‐omics analysis

The quality control, batch correction, cell clustering and annotation of scRNA‐seq profiles were performed according to usual routines^23^. The detailed procedure is elaborated in Supplementary methods 1.1. We used the ‘STRINGdb’ R package^24^ to construct a protein–protein interaction (PPI) network using genes from the intersection of differentially expressed genes (DEGs) from bulk RNA‐seq and marker genes for exhausted T cells. The “GSVA” package was used to convert gene expression profiles into pathway activity scores (Tables S3 and S4), with pathway data sourced from the Molecular Signatures Database (MSigDB) (https://www.gsea‐msigdb.org/gsea/msigdb).^25^ The R package ‘CellChat’^26^ enables cell interaction analysis in scRNA‐seq data to uncover communication networks between cells (Figure S1). We used SCENIC^27^ to infer the core transcription factors (TFs) in T‐cell clusters. Monocle2^28^ was used for trajectory analysis of T‐cell clusters. T‐cell clusters were subjected to dimensionality reduction, standardization, clustering and visualization (detailed in Supplementary methods 1.2) (Figures S1 and S2).

### 2.4 Constructing a pseudo‐bulk set using scRNA‐seq data

To process the scRNA‐seq data matrix X, where rows represent cells and columns represent genes, we conducted a weighted random sampling of n blocks. For the input data, we underwent a rigorous data preprocessing process. First, we used Seurat's NormalizeData function to standardize the raw data. Then, we used the FindVariableFeatures function to identify highly variable genes. Finally, we scaled the data using ScaleData.^25^ Within each block, the average gene expression levels of the cells were calculated to form a row in the pseudo‐bulk matrix. Here, we established a sampling size of 100 and carried out a total of 1000 samplings. Simultaneously, for each block, we chose the most frequently occurring label as the label for the current pseudo‐bulk. Notably, the sampling weights were adjusted so that the current cell type being sampled was weighted 10 times more than other types. Ultimately, we derived the pseudo‐bulk matrix and its corresponding pseudo‐bulk labels $Y_{2}$.

### 2.5 Aligning data using the domain adaptive

Next, we introduced a domain adaptation model for aligning the distributions between pseudo‐bulk and real‐bulk data. We constructed an end‐to‐end model in which the AE component takes the bulk matrix $X_{1}$ as input. The bulk data was also subjected to 1000 rounds of sampling to expand the sample size. This was accompanied by log2 normalization and only the retention of samples with survival information, aiming to capture the distribution of the real‐bulk data:

(1) $$Y_{1}=\text{Relu}\left(X_{1}W^{\left(1\right)}+b^{\left(1\right)}\right)$$

(2) $$Z_{1}=\text{Sigmoid}\left(Y_{1}W^{\left(2\right)}+b^{\left(2\right)}\right)$$

Here, $Y_{1}$ depicts the feature representation extracted by the AE, and $Z_{1}$ is the output of the model. For prediction, the CE part takes the pseudo‐bulk matrix $X_{2}$ as input:

(3) $$Y_{2}=\text{Relu}\left(X_{2}W^{\left(1\right)}+b^{\left(1\right)}\right)$$

(4) $$Z_{2}=\mathrm{TSS}\left(Y_{2}W^{\left(2\right)}+b^{\left(2\right)}\right)$$

Here, $Y_{2}$ denotes the features extracted by the CE and $Z_{2}$ represents the scores predicted by the model (Tables S8–S11). TSS refers to the temperature‐scaled sigmoid:

(5) $$\mathrm{TSS}\left(x\right)=\frac{1}{1+e^{\frac{-x}{t}}}$$

Based on the AE and CE components, we have constructed the corresponding loss functions:

(6) $$L_{\text{reconstruction}}=\lambda_{2}\sqrt{\frac{1}{N}\sum_{i=1}^{N}{\left(z_{i}-\hat{z_{i}}\right)}^{2}}$$

(7) $$L_{\text{category}}=\lambda_{1}\sum_{i=1}^{N}\sum_{j=1}^{C}z_{i,j}\cdot \log\left(\hat{z_{i,j}}\right)$$

where $\lambda_{1}$ and $\lambda_{2}$ are both set to 0.1. To align the low‐dimensional feature distributions between the source domain (pseudo‐bulk) and the target domain (real‐bulk), we construct an MMD loss for the feature maps extracted by AE and CE:

(8) $$L_{\mathrm{MMD}\left(Y_{1},Y_{2}\right)}=\lambda_{3}{\left|\frac{1}{n_{Y_{1}}}\sum_{i=1}^{n_{Y_{1}}}\phi \left(x_{i}^{Y_{1}}\right)-\frac{1}{n_{Y_{2}}}\sum_{j=1}^{n_{Y_{2}}}\phi \left(x_{j}^{Y_{2}}\right)\right|}^{2}$$

where $n_{Y_{1}}$ and $n_{Y_{2}}$ represent the number of features extracted from AE and CE, respectively, $x_{i}^{Y_{1}}$ and $x_{j}^{Y_{2}}$ are samples drawn from $Y_{1}$ and $Y_{2}$ respectively, with $\lambda_{3}$ set to 0.8. The feature mapping function $\phi \left(\cdot \right)$ used here is a Gaussian kernel function, with the specific form as follows:

(9) $$\phi \left(x\right)=\frac{1}{\sqrt{2\sigma^{2}}}\exp\left(\frac{-{\left|x-\mu \right|}^{2}}{2\sigma^{2}}\right)$$

The final loss function is constructed as follows:

(10) $$L=L_{\text{reconstruction}}+L_{\text{category}}+L_{\mathrm{MMD}\left(Y_{1},Y_{2}\right)}$$

Through experimentation, we eventually used a learning rate of 1e−3, set the intermediate feature dimensions of the AE and CE models to 256, and trained the model for 100 epochs with a batch size of 100.

### 2.6 Predicting T‐cell exhaustion in CRC patients using knowledge distillation

We combined the trained AE and CE models to construct the teacher model. The specific steps involve using the AE model to obtain an intermediate feature representation $Y_{1}$ by inputting the real bulk matrix $X_{1}$. Then, the CE model outputs soft labels $Z_{2}$ using $Y_{1}$ as an intermediate feature input. (The CE model predicts scores for different TEX stages, and we only used the scores for the final TEX stage as the soft label output of the model.) We used the GSVA software to convert the real bulk matrix $X_{1}$ into a pathway activity matrix $X_{3}$ for use as input for the student model (or the original bulk matrix and other functional set activity matrices). The construction of the student model is a multi‐layer neural network designed to predict the T‐cell exhaustion score $\hat{Z}$:

(11) $$\hat{Z}=\text{Sigmoid}\left(\text{Relu}\left({\left(X_{3}W^{\left(1\right)}+b\right)}^{\left(1\right)}\right)W^{\left(2\right)}+b^{\left(2\right)}\right)$$

In this process, we apply an L1 regularization constraint to the weight matrix $W^{\left(1\right)}$, aimed at selecting pathways or genes related to exhausted T cells, L1 regularization was implemented by setting the kernel regularizer parameter to L1 (1e‐4).

Through experimentation, we eventually used a learning rate of 1e‐3, set the intermediate feature dimensions to 128, and trained the model for 4000 epochs with a batch size of 100. Notably, to enhance the stability of the model, we used bootstrap techniques to use the mean of multiple runs as the final model output. To ensure the reproducibility of the model, we set fixed random seeds for each stage of model training.

### 2.7 Using exhaustion score for survival analysis

The exhaustion scores (Table S11) obtained from the model are divided into high and low groups based on the median. We used the ‘survival’ package for conducting survival analysis. Nomogram plots are commonly used for visualizing and interpreting complex statistical models to gain a more intuitive understanding of the impact of specific factors on a particular outcome. Using the ‘rms’ package, we created nomogram plots for this purpose.

### 2.8 Analysis of TEX pathway activity

The TEX signalling pathway features and signature gene sets are sourced from the MSigDB. The ‘GSVA’ R package is used to estimate the TEX pathway activity scores for each patient (Tables S9 and S10). Consistency clustering is performed using the ‘ConsensusClusterPlus’ R package, while the ‘ComplexHeatmap’ package is used for visualization purposes.

### 2.9 Immune infiltration analysis

CIBERSORT is a tool for computing the composition of infiltrating immune cells. It estimates the relative abundance of different cell types in complex tissue samples using a deconvolution method. To analyse the immune microenvironment in patients with CRC, CIBERSORT was used to compute the composition of infiltrating immune cells. ESTIMATE can be used to approximate the relative proportions of immune cells and stromal cells in tumour tissue. To quantify stromal cell activity in CRC, ESTIMATE is used to calculate the stromal score.

### 2.10 Drug sensitivity analysis

oncoPredict^29^ is a widely used tool to predict the sensitivity of patients to different treatment options by integrating tumour genomic data. We used oncoPredict for drug sensitivity analysis. The specific steps involve dividing the predicted T‐cell exhaustion scores into two groups based on the median and using *t*‐tests to identify groups with significantly different means. This approach provides in‐depth insights into the tumour's response to specific drugs. R version 4.3.0 was used for statistical analyses. Kaplan–Meier curves were used to compare overall survival (OS).

### 2.11 Statistical analysis

The Wilcoxon rank‐sum test was used to assess the significance between variables. A *p*‐value of less than 0.05 was considered statistically significant. Cox regression was applied to construct prognostic models. The *t*‐test was used to determine drugs with significantly different mean IC50 values.

## 3 RESULTS

### 3.1 Accuracy and prognostic value of DeepTEX in T‐cell exhaustion identification and risk scoring in CRC

We validated the effectiveness and generalization capability of the DeepTEX model using both newly generated pseudo‐bulk data and a set of newly added bulk data. For the pseudo‐bulk data at different stages of T‐cell exhaustion, we used performance metrics such as area under the receiver operating characteristic curve (AUROC) and area under the precision‐recall curve (AUPRC) to validate the domain adaptation model (Figure S3). For the bulk dataset, we used a pre‐trained domain adaptation model to predict T‐cell exhaustion scores. DeepTEX, a state‐of‐the‐art deep learning modal, was used to discern the state of T‐cell exhaustion, yielding an AUC of 0.92 in ROC curve analysis (Figure S3A) and an AUC of 0.93 in precision‐recall curve analysis (Figure S3B), confirming its high precision and recall performance.

We validated the ability of the DeepTEX model to perform survival analysis on CRC patients from the gene, hallmark, KEGG and teacher model perspective, demonstrating its significant advantage over single‐modal methods in analysing T‐cell exhaustion in CRC (Figure S3E). On comparing the prognostic survival of CRC patients to existing methods CIBRTSORT and GSVA, we found that DeepTEX achieved a higher significance (Figure S4). We also used the DeepTEX model to predict key genes associated with cancer T‐cell exhaustion, identifying FLT3LG and XCL1 as potential biomarkers with statistically significant correlations in Kaplan–Meier survival curve analysis (Figure S6).^30^, ^31^ These results highlight the superior accuracy of DeepTEX in identifying T‐cell exhaustion compared to other methods.

In summary, our findings confirm the superiority of DeepTEX as a robust tool for TEX identification in CRC, advancing the field towards more precise and effective cancer immunotherapies. The performance of the DeepTEX model for identifying T‐cell exhaustion and its comparative analysis with CIBERSORT and GSVA are detailed in the Supplementary Material (Sections 2.1–2.3) (Figure S5).

### 3.2 Single‐cell analysis of CRC liver metastases

**FIGURE 2:**
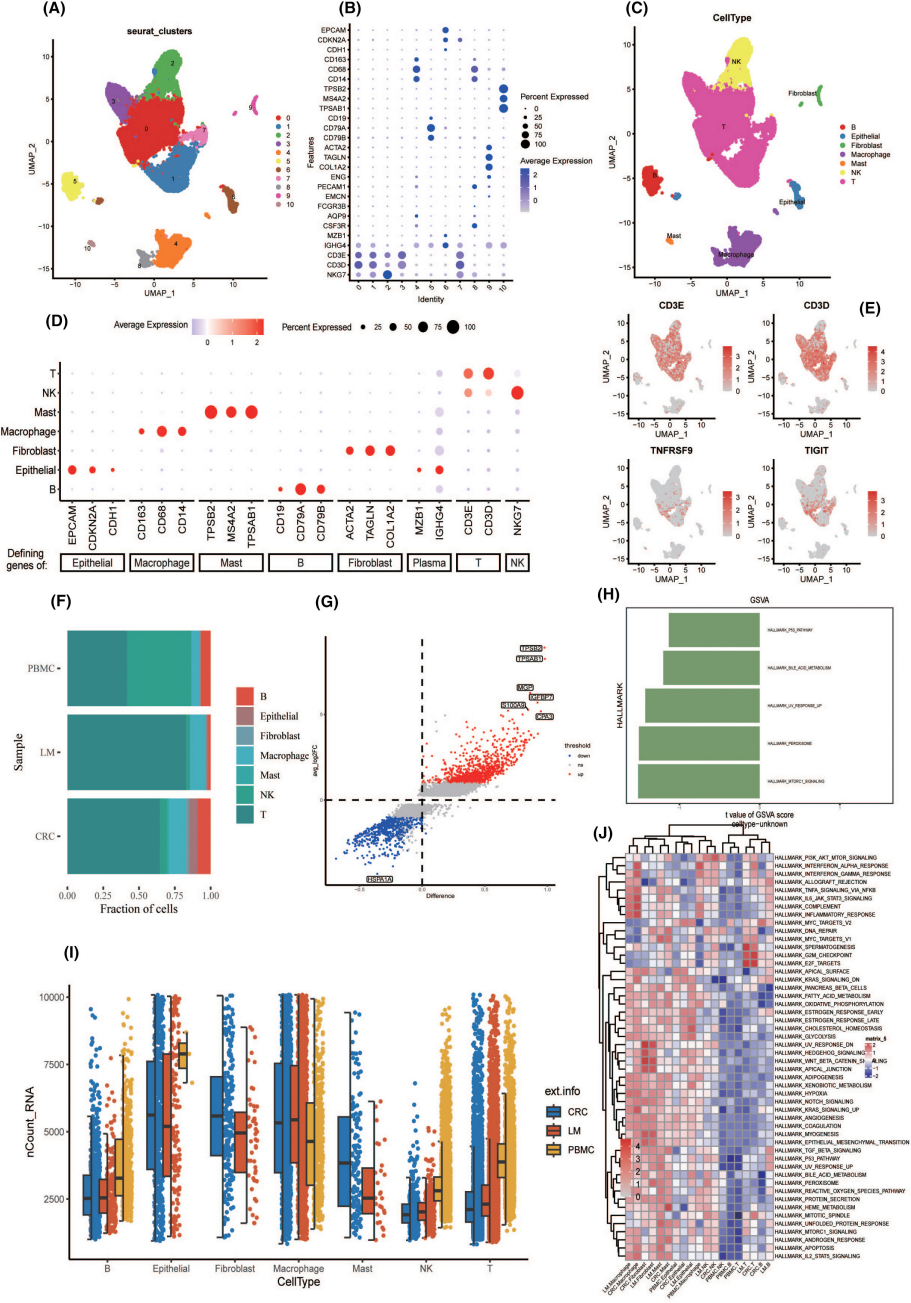
Single‐cell analysis of CRC liver metastases. (A) Classify cells into 11 clusters. (B) The average expression of representative marker genes for the main cell clusters in the integrated human CRC data. (C) Eleven cell clusters are annotated as seven cell types. (D) The expression of marker genes corresponding to each cell type in CRC single‐cell data. (E) CD3E and CD3D exhibit the most significant expression in CRC T cells. (F) The cell proportions of seven cell types in three samples. (G) Significantly downregulated and upregulated genes. (H) GSVA presents five pathways differentiating metastatic and primary sites. (I) The number of T cells is higher in metastatic cancer LM, PBMC than in primary CRC. (J) Pathways with high activity in both metastatic and primary sites.

To explore the regulatory mechanisms of T‐cell exhaustion in primary CRC and metastatic cancer in greater detail, we clustered cells and visualized the cell clusters using UMAP (Figure 2A). This analysis was based on the dot plot of the average expression of representative marker genes in the main cell clusters of our integrated human CRC data (Figure 2B). We manually annotated the 11 cell clusters as seven cell types, which included B cells, epithelial cells, fibroblasts, macrophages, mast cells, natural killer (NK) cells and T cells (Figure 2C). We tested the accuracy of our cell type labelling by examining the expression of recognized cell markers in the seven cell types from previous research (Figure 2D). In the annotated T‐cell clusters, CD3E and CD3D are significantly enriched.^32^ At the same time, we also observed the expression of T‐cell exhaustion markers (TNFRSF9 and TIGIT) (Figure 2E–J).^33^

### 3.3 In‐depth analysis of T‐cell exhaustion mechanisms in primary CRC and liver metastases

**FIGURE 3:**
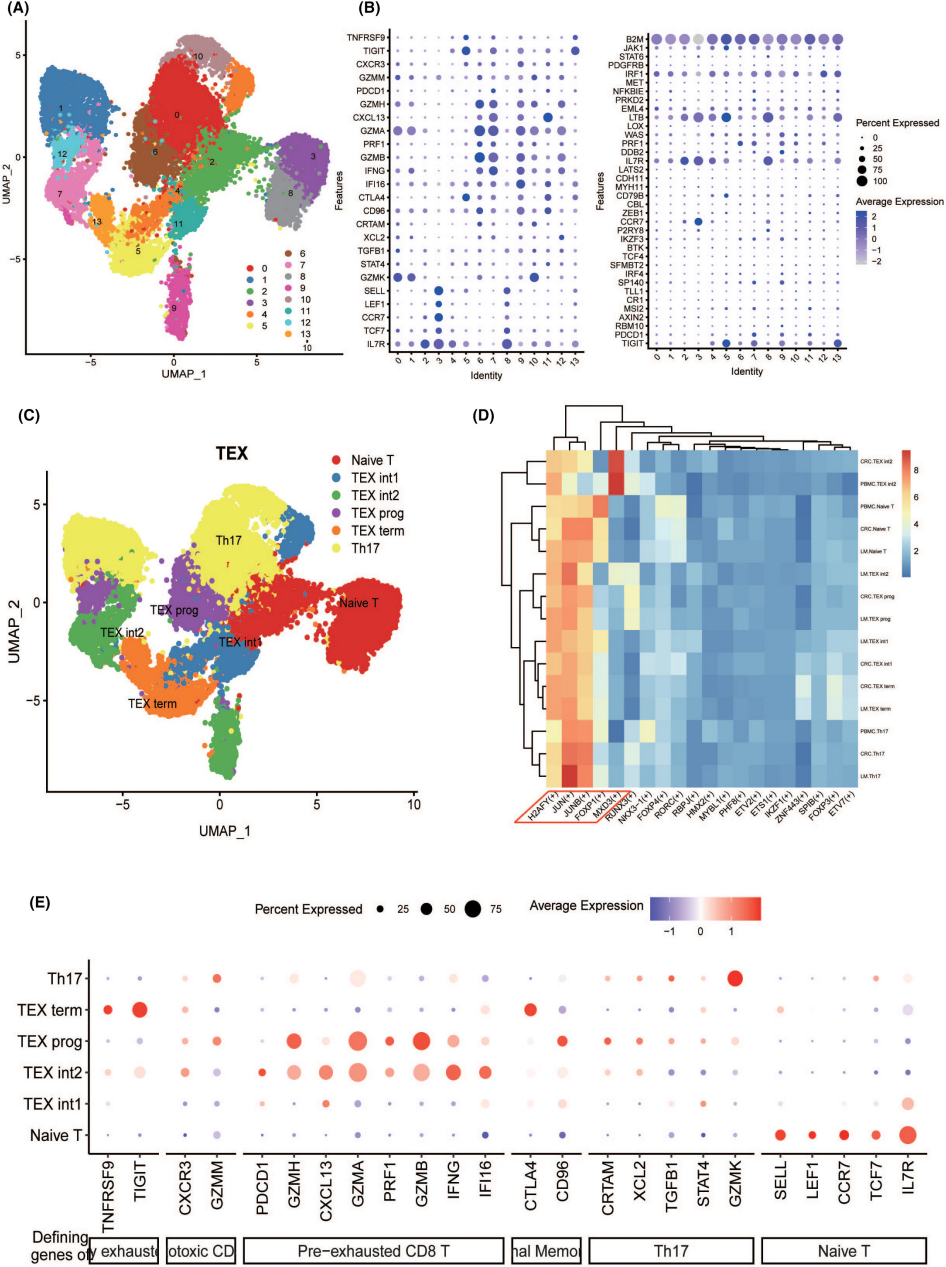
In‐depth analysis of T‐cell exhaustion mechanisms in primary CRC and liver metastases. (A) Cells were classified into 14 clusters. (B, C) The average expression of representative marker genes of major cell clusters associated with T‐cell exhaustion in integrated human CRC data. (D) The top 20 TFs activities in different CRC types. (E) Six functional cell types related to the degree of T‐cell exhaustion.

Eleven clusters were manually annotated as seven cell types, which included B cells, epithelial cells, fibroblasts, macrophages, mast cells, NK cells and T cells. From the annotation results, a significant enrichment of T cells was observed, indicating a higher degree of immune infiltration in patients with CRC. To further explore the regulatory mechanisms of T‐cell exhaustion in primary CRC and liver metastases, we selected T cells for sub‐class annotation and analysis, visualizing T‐cell clustering using UMAP (Figure 3A). Naive T cells were annotated through the expression of marker genes (Figure 3B–E).^34^

### 3.4 Downstream validation of a deep learning‐based multi‐omics integration model

**FIGURE 4:**
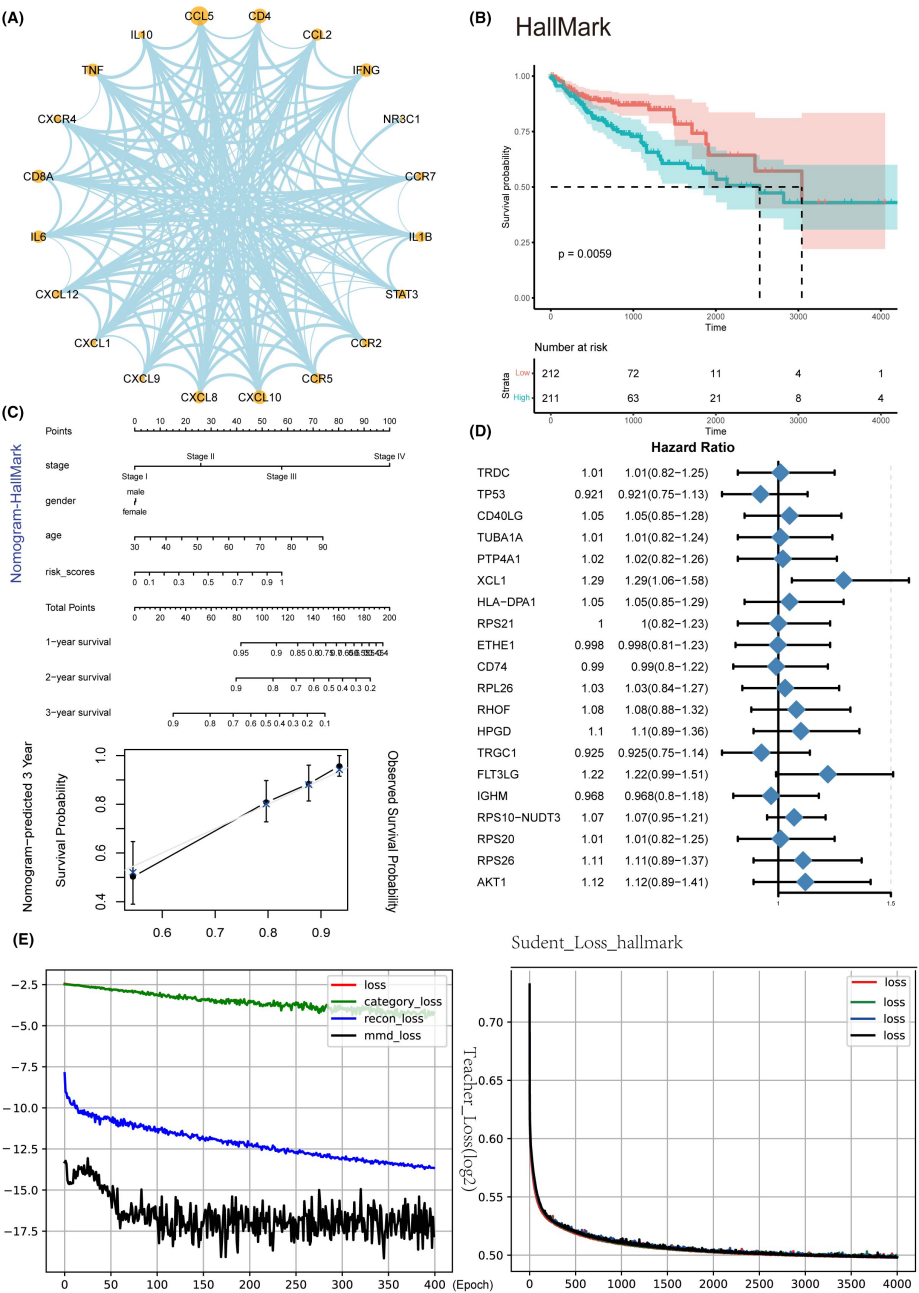
Downstream validation of a deep learning‐based multi‐omics integration model. (A) The protein–protein interaction network. (B) The prognostic significance of Hallmark. (C) The consistency between predicted and observed 3‐year survival rates. (D) Certain genes may be significant risk factors in CRC. (E) Losses of the teacher model and the student model are discussed.

DeepTEX then constructs a PPI network through the STRING database, showcasing the top 20 proteins (Figure 4A). The protein interaction network is built using genes associated with TEX, with all T‐cell marker genes used to expand the gene set used by the deep learning model. We found that the CXCL, CCL and CCR protein families, along with classical star proteins such as IFNG and STAT3, play significant roles in the development of CRC. Previous research has shown that macrophage‐secreted C‐C motif chemokine ligand 5 (CCL5) stabilizes PD‐L1 in vitro and in vivo, promotes immune escape, potentially offering therapeutic and prognostic significance for human cancers.^35^, ^36^ The interaction between CXCL and CCR protein families and the interleukin (IL) family of inflammatory factors plays a significant role in T‐cell exhaustion in primary and metastatic CRC.^37^, ^38^, ^39^ The TEX score predicted by DeepTEX was divided into the high‐risk and low‐risk groups according to the median. The survival rate of high‐risk and low‐risk patients with TCGA showed significant differences, revealing the potential value of TEX score in the prognosis of patients (Figure 4B). Simultaneously, the activity matrix obtained by using different functional gene sets, such as GSVA pathway activity matrix, the high and low TEX groups can also be well distinguished. The nomogram indicates the prognostic relevance of high versus low TEX scores, establishing a nomogram of TEX scores and clinical factors in the TCGA cohort. The calibration plot of the nomogram shows the consistency between predicted and observed 3‐year survival rates, evaluating the accuracy of the DeepTEX model in predicting the probability of clinical outcomes through calibration (Figure 4C). The hazard ratio reveals FLT3LG and XCL1 as potential risk factors for CRC (Figure 4D).^40^ We also found that the loss of both the teacher and student models rapidly decreases during the training phase (Figure 4E). In the student model, we used boosting techniques to enhance model robustness, with the loss transformations of multiple sub‐models showing high overlap.

### 3.5 Immune cell infiltration analysis in CRC

**FIGURE 5:**
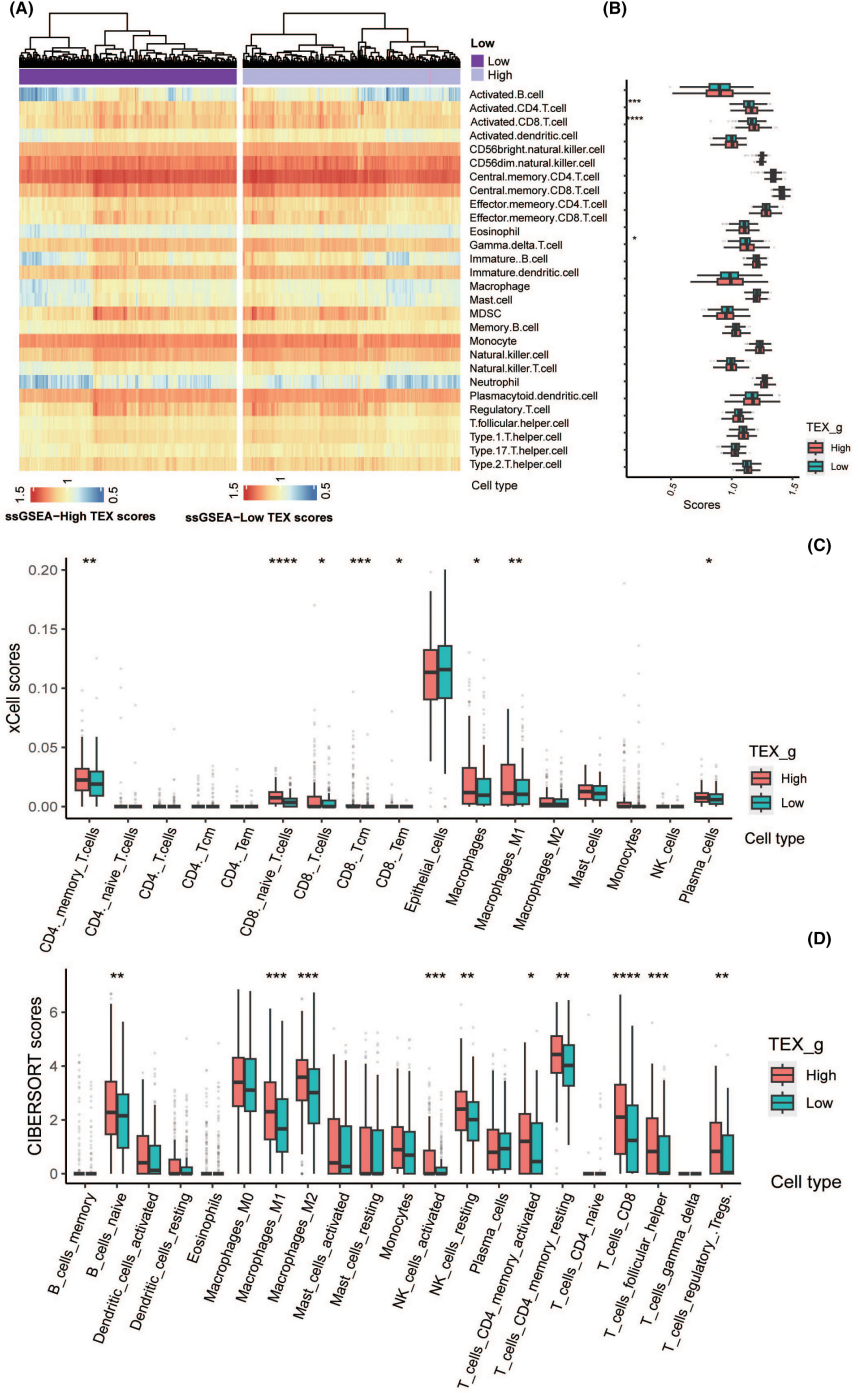
Immune infiltration analysis in CRC. (A, B) T‐cell exhaustion score high and low immune cell infiltration differences. (C) xCell chart shows the proportion of 17 immune cell subgroups in high and low exhaustion score groups. (D) CIBERSORT chart shows the proportion of 21 immune cell subgroups in high and low exhaustion score groups. ****p < 0.0001, ***p < 0.001, **p < 0.01, *p < 0.05.

Immune cell infiltration plays an important role in the development and prognosis of cancer. We explored immune cell infiltration in samples with high and low T‐cell exhaustion from both bulk and single‐cell data. In bulk data, immune cell infiltration was quantified using the ssGSEA (Single‐Sample Gene Set Enrichment Analysis) algorithm. Samples with high T‐cell exhaustion from TCGA showed greater immune cell infiltration, such as activated CD4+ T cells, activated CD8+ T cells, effector memory CD4+ T cells, effector memory CD8+ T cells and NK cells. Immune cell infiltration in the high and low TEX score sample groups was explored by integrating TCGA bulk data and single‐cell data. In the integrated data, immune infiltration was quantified using the ssGSEA algorithm. More immune cell infiltration was shown in both high and low T‐cell exhaustion score CD4+, CD8+ T‐cell samples (Figure 5A,B). Combining immune cell infiltration results from bulk and single‐cell data revealed that the high TEX risk group exhibited more prominent immune cell infiltration, especially CD4+, CD8+ T cells (Figure 5C). Integrating bulk samples, we used the ‘CIBERSORT’ programme to calculate the relative abundance of 21 different immune cell types (Figure 5D) and also observed a notably higher infiltration level of T cells in the high TEX risk group.

### 3.6 Functional mechanism analysis of T‐cell exhaustion in CRC

**FIGURE 6:**
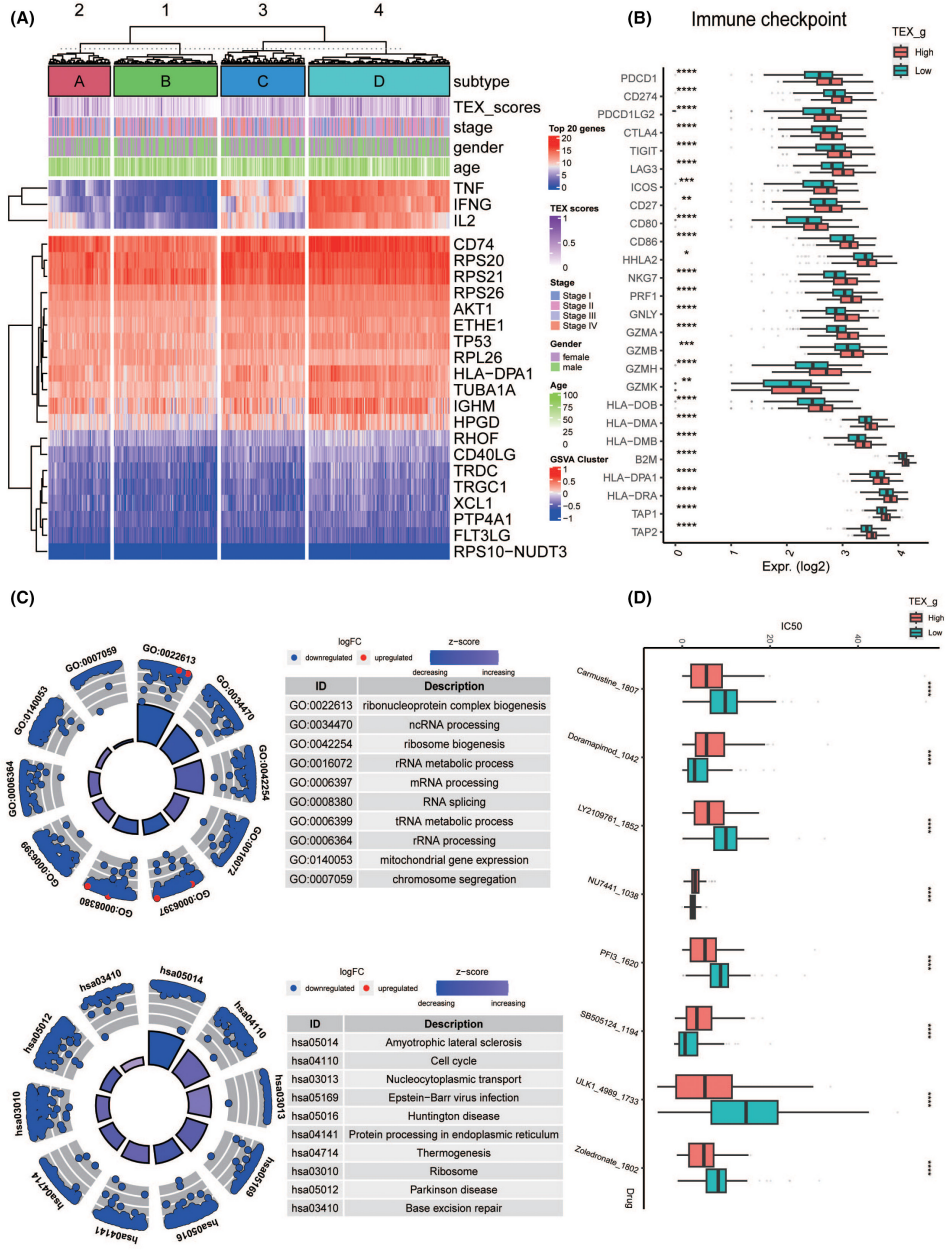
Analysis of T‐cell exhaustion functional mechanisms in CRC. (A) The stages of T‐cell exhaustion and the T‐cell exhaustion scores predicted by the model. (B) Expression of immune checkpoints in relation to high and low T‐cell exhaustion scores. (C) Enrichment results in a circular format, along with the names of functional pathways. (D) T‐cell exhaustion scores of patients correlate with sensitivity to eight types of drugs. ****p < 0.0001, ***p < 0.001, **p < 0.01, *p < 0.05.

The GSVA cluster heatmap illustrates the stages of T‐cell exhaustion (Figure 6A). It displays the expression of differentially expressed functional marker genes post‐integration into TCGA and scRNA‐seq data, along with associated clinical features and the extent of T‐cell exhaustion. The results indicate significant variations in the expression of TEX‐related marker genes, such as RPS20 and HLA‐DPA1, across different exhaustion stages (Figure 6A,B).^41^ Moreover, we have observed immune checkpoints in varying levels of T‐cell exhaustion. Most checkpoints showed a significantly higher expression in the low TEX score group compared to the high TEX score group, suggesting potential targets for immune checkpoint inhibitors. To explore the potential functions of crucial TEX genes, we conducted downstream gene ontology (GO) and Kyoto Encyclopedia of Genes and Genomes (KEGG) functional enrichment analyses, and revealed that pathways such as ribonucleoprotein complex biogenesis, cell cycle, and nucleocytoplasmic transport play key roles in the development of CRC (Figure 6C). Finally, we performed a drug sensitivity analysis based on the high and low T‐cell exhaustion scores in CRC and provided a list of potential drugs related to the TEX (Figure 6D).

## 4 DISCUSSION

With the development of scRNA‐seq technology, cell heterogeneity, especially that of immune cells and their regulatory mechanisms, has been widely studied in many complex diseases.^42^ For example, regulatory T cells (Tregs) wield a pivotal immunological influence within the TME.^43^ Fibroblasts can influence outcomes in cardiomyopathy such as myocardial fibrosis.^44^ Immune models have been constructed through high‐throughput sequencing data generated by single‐cell technology, which could provide a potential target for the diagnosis and treatment of major diseases.^45^ Simultaneously, the regulatory mechanism of immune cells in the TME has been confirmed through biological experiments.^46^ In this study, we used bulk and single‐cell CRC and liver metastasis data to explore the heterogeneity of T‐cell exhaustion. T‐cell exhaustion in late‐stage CRC and CRC liver metastasis compromises immunity and results in a generally poor prognosis. The targeting of T cells is gaining prominence in the field of cancer immunotherapy. This study aims to develop a novel deep learning algorithm, DeepTEX, through a teacher–student model integrating scRNA‐seq and bulk RNA‐seq data generated by high‐throughput experiments for assessing T‐cell exhaustion in CRC patients. We conducted a comprehensive single‐cell and bulk analyses on primary CRC and metastatic samples, categorizing T cells into four distinct stages: TEXprog, TEXint1, TEXint2, TEXterm (Figure S1A–E). Additionally, we used ‘CellChat’ to explore potential communication between T cells and other cell types in CRC and CRC liver metastasis, identifying frequent interactions between T cells and fibroblasts as well as macrophages (Figure S2). Through the analysis of TFs activity in T cells, the activity of H2AF1 is significantly higher in various stages of T‐cell exhaustion than in the non‐exhausted stage, whereas JUN showed lower activity during T‐cell exhaustion. Additionally, MXD3 exhibited significantly high activity in primary and metastatic T‐cell exhaustion stages. TFs such as H2AF1, JUN and MXD3 may serve as potential biomarkers in CRC diagnosis and treatment.^47^, ^48^, ^49^ In addition, through protein interaction analysis, we found important protein families such as CXCL, CCL and CCR, which regulate immunity, angiogenesis, stem cell trafficking, cell growth and organ‐specific metastasis in the TME T‐cell exhaustion process.^50^

In the knowledge distillation stage of DeepTEX, we achieved interpretable biological node screening by inputting different modalities (such as gene and pathway activity matrices) as node representations and adding sparsity constraints to the first layer of the student model. As a result, we identified genes and pathways associated with T‐cell exhaustion, such as the gene FLT3LG and XCL1 (Figure S6A,B) and pathway HALLMARK_TNFA_SIGNALING_VIA_NFKB^51^ (Tables S6 and S7). Using genes and pathways as predictive features, we drew Kaplan–Meier curves based on T‐cell exhaustion scores and observed whether the DeepTEX prediction results could effectively segregate patients with high and low survival rates, thereby assessing both the accuracy and reliability of the model. Through survival analysis using the patient's T‐cell exhaustion score predicted by DeepTEX, we found significant differences in the survival curves divided by the median. We also used the GSE159216 CRC dataset and demonstrated through survival analysis that DeepTEX can accurately predict T‐cell exhaustion scores and clearly classify patient survival (Figure S6). By using the visual representation of the Kaplan–Meier curve, we showed that DeepTEX can provide valuable insights to predict the level of T‐cell exhaustion in CRC.

DeepTEX uses deep learning methods to integrate CRC scRNA‐seq and bulk RNA‐seq data, enabling the revelation of T‐cell exhaustion heterogeneity in CRC patients. This strategy not only uncovers the relationship between T‐cell exhaustion and tumour prognosis but also elucidates its role in immunology. By examining T‐cell exhaustion levels and their prognostic value in primary CRC and liver metastasis, DeepTEX reveals key regulatory mechanisms involved in the development and progression of primary and metastatic tumours. This comprehensive understanding of the TME in primary CRC and CRC liver metastasis is crucial. Currently, spatial transcriptomics data predominantly focus on cell–cell communication in neural tissues. This study has not yet integrated spatial transcriptomics data related to CRC and metastatic cancer to explore the spatial interactions of T‐cell exhaustion within CRC. In the future, we aim to combine DeepTEX with spatial omics data to provide potential biomarkers for clinical treatment, aiding in the development of more precise therapeutic strategies. For instance, identifying ligand‐receptors, signalling proteins and critical pathways that mediate cell–cell interactions in spatial contexts can reveal mechanisms underlying tumour development and metastasis. This integration offers new perspectives for the clinical treatment of CRC and innovative immunotherapy strategies.

## AUTHOR CONTRIBUTIONS

**Mingcong Xu:** Conceptualization (equal); data curation (equal); formal analysis (equal); visualization (equal); writing – original draft (equal); writing – review and editing (equal). **Guorui Zhang:** Data curation (equal); project administration (equal); writing – original draft (equal); writing – review and editing (equal). **Ting Cui:** Investigation (equal); visualization (equal). **Jiaqi Liu:** Methodology (equal); validation (equal). **Qiuyu Wang:** Funding acquisition (equal); writing – review and editing (equal). **Desi Shang:** Formal analysis (equal); resources (equal). **Tingting Yu:** Formal analysis (equal); resources (equal). **Bingzhou Guo:** Formal analysis (equal); visualization (equal). **Jinjie Huang:** Methodology (equal); project administration (equal); writing – review and editing (equal). **Chunquan Li:** Conceptualization (equal); funding acquisition (equal).

## FUNDING INFORMATION

This work was supported by National Natural Science Foundation of China [62171166, 62272212, 62272211]; The Science and Technology Innovation Talent Program of Hunan Province of China [2024RC1062]; Research Foundation of the First Afffliated Hospital of University of South China for Advanced Talents [20210002–1005 USCAT‐2021‐01]; Natural Science Foundation of Hunan Province [2023JJ30536, 2023JJ30535]; Clinical Research 4310 Program of the University of South China [20224310NHYCG05]. Funding for open access charge: National Natural Science Foundation of China [62171166, 62272212, 62272211]; The Science and Technology Innovation Talent Program of Hunan Province of China [2024RC1062]; Research Foundation of the First Afffliated Hospital of University of South China for Advanced Talents [20210002–1005 USCAT‐2021‐01]; Natural Science Foundation of Hunan Province [2023JJ30536, 2023JJ30535]; Clinical Research 4310 Program of the University of South China [20224310NHYCG05].

## CONFLICT OF INTEREST STATEMENT

The authors declare that there are no potential conflicts of interest.

## Supporting information


**Figure S1.** **|** Liver metastasis in CRC is associated with a higher degree of T‐cell exhaustion. (A) Box plots show the number of cells at different stages of T‐cell exhaustion in primary CRC, metastatic cancer and PBMCs. Liver metastasis exhibits a higher degree of terminal exhaustion compared to primary CRC. (B) Expression of IFNG, PDCD1, TNFRSF9 and TIGIT at various stages of T‐cell exhaustion. (C) Calculation of highly variable genes between primary CRC and liver metastasis, blood metastasis. (D) Monocle single‐cell pseudotime trajectory analysis for primary CRC and liver metastasis, blood metastasis. (E) GSVA for primary CRC and liver metastasis.

**Figure S2.** | Analysis of malignant cells in CRC. (A) Dentifying large‐scale chromosomal copy number variations by InferCNV. (B) The box plots illustrate CNV scores, with epithelial cells exhibiting the highest scores, indicating their heightened susceptibility to transforming into malignant tumour cells. (C) The circle plot illustrates the communication network among immune cells, epithelial cells and fibroblasts in CRC. (D) The heatmap displays the number and intensity of interactions between immune cells, epithelial cells and fibroblasts. (E) Display and calculate the intensity of ligand–receptor interactions, and demonstrate the specific communication between cells through specific ligand–receptor interactions. (F) Heatmap shows the cell interaction strength of IFN‐II signalling network.

**Figure S3.** | Performance Evaluation of DeepTEX in T‐cell exhaustion identification. (A) The diagnostic efficacy of DeepTEX was assessed through ROC curve analysis, which yielded an AUC of 0.92. (B) The precision‐recall curve analysis demonstrated an AUC of 0.93 (C) The boxplot demonstrates that DeepTEX achieves AUROC and AUPRC values greater than 0.9 for identifying exhausted T cells. (D) The histogram illustrates the significance of survival differences observed in the algorithm survival analysis. For predicting T‐cell exhaustion using KEGG, HALLMARK and GENE signatures, DeepTEX achieved (−log10) *p*_value of 2.6021, 2.2218 and 1.7421, respectively, compared to 0.0269 for CIBERSORT and 0.7418 for GSVA. (E) Line chart shows the performance of DeepTEX in significance of survival analysis differences and run time compared with other models.

**Figure S4.** | Survival Analysis Validation for DeepTEX. (A–D) DeepTEX performs survival analysis on CRC patients from the perspectives of Gene, Hallmark, KEGG and Teacher model. (E, F) The Kaplan–Meier curves for overall survival rates of T‐cell exhaustion in CRC using GSVA and CIBERSORT methods. ***p < 0.001, **p < 0.01, *p < 0.05.

**Figure S5.** | Risk Scores for T‐cell exhaustion in CRC Using DeepTEX, GSVA and CIBERSORT Nomograms. (A–D) DeepTEX performs risk scores on CRC patients from the perspectives of Gene, Hallmark, KEGG and Teacher model. (E, F) The nomograms for risk scores of T‐cell exhaustion in CRC using GSVA and CIBERSORT methods.

**Figure S6.** | Validation of the DeepTEX (A, B) the prognostic significance of FLT3LG and XCL1. (C–F) GSE159216 CRC dataset to comprehensively evaluate the capability of the DeepTEX model in predicting T‐cell exhaustion related genes in CRC, across four distinct aspects: Gene, Hallmark, KEGG and Teacher model. ***p < 0.001, **p < 0.01, *p < 0.05.

**Table S1.** Summary statistics of all datasets enrolled in this work.
**Table S2.** TCGA_CRC Patients clinical information.
**Table S3.** HALLMARK‐GSVA matrix.
**Table S4.** KEGG‐GSVA matrix.
**Table S5.** Gene importance score.
**Table S6.** HALLMARK importance score.
**Table S7.** KEGG importance score.
**Table S8.** Risk score (TCGA bulk data as model input).
**Table S9.** Risk score (HALLMARK‐GSVA as model input).
**Table S10.** Risk score (KEGG‐GSVA as model input).
**Table S11.** Deconvolution score of T‐cell exhaustion stage.
**Table S12.** Teacher network risk scores of DeepTEX.
**Table S13.** Data collection information comparison.

Data S1:

## Data Availability

The data and source code generated in this study was deposited in GitHub [https://github.com/TOSTRING‐Z/DeepTEX], which was implemented using Python, and Pytorch.
