# Supplementary figures and images for: Cross‐modal integration of bulk RNA‐seq and single‐cell RNA sequencing data to reveal T‐cell exhaustion in colorectal cancer
Source: J Cell Mol Med. 2024 Sep 29;28(18):e70101. doi: 10.1111/jcmm.70101 (PMC11439987; doi:10.1111/jcmm.70101)

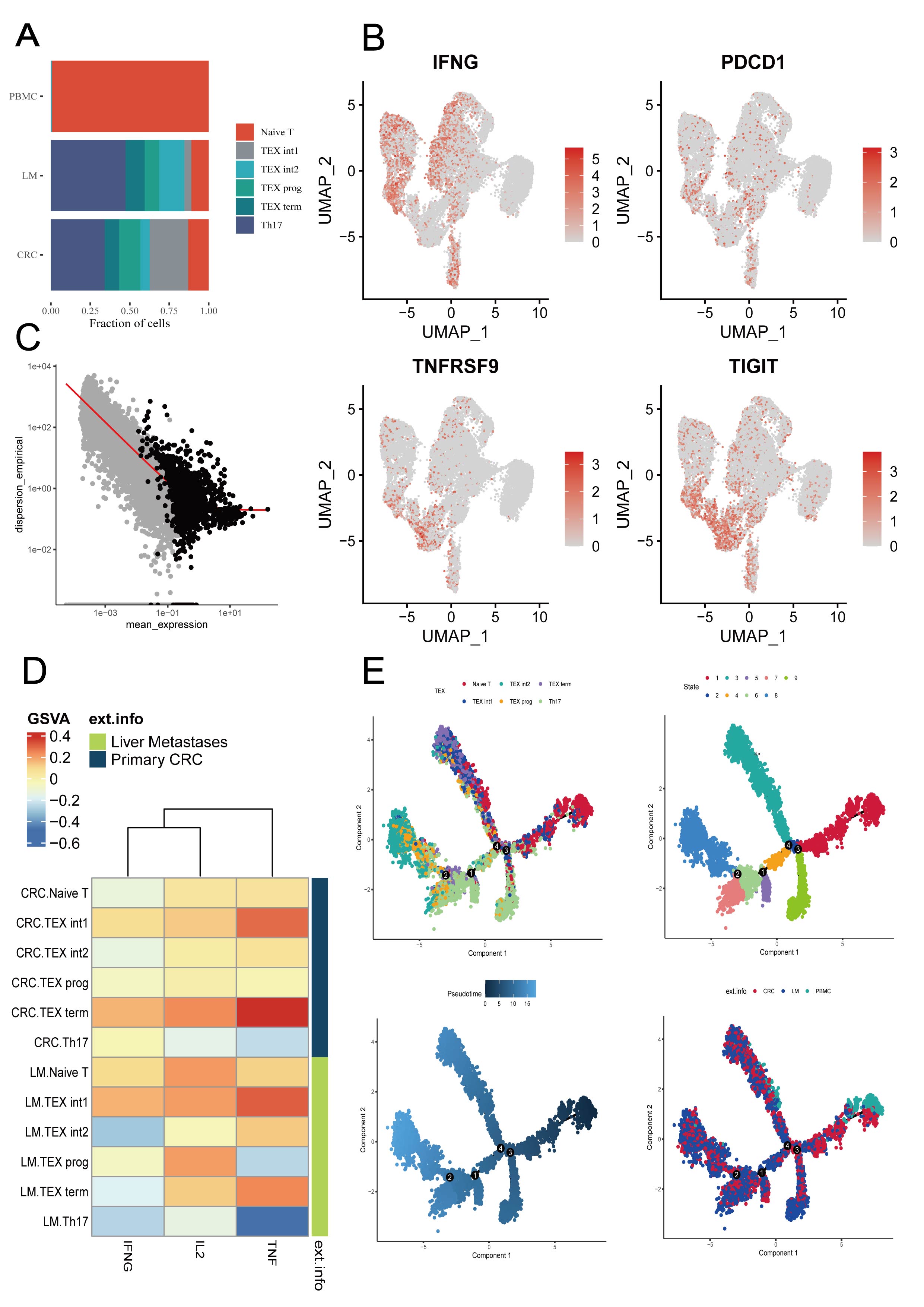

Supplement: Supplementary file 1 — Figure S1. | Liver metastasis in CRC is associated with a higher degree of T‐cell exhaustion. (A) Box plots show the number of cells at different stages of T‐cell exhaustion in primary CRC, metastatic cancer and PBMCs. Liver metastasis exhibits a higher degree of terminal exhaustion compared to primary CRC. (B) Expression of IFNG, PDCD1, TNFRSF9 and TIGIT at various stages of T‐cell exhaustion. (C) Calculation of highly variable genes between primary CRC and liver metastasis, blood metastasis. (D) Monocle single‐cell pseudotime trajectory analysis for primary CRC and liver metastasis, blood metastasis. (E) GSVA for primary CRC and liver metastasis. [file JCMM-28-e70101-s004.jpg]

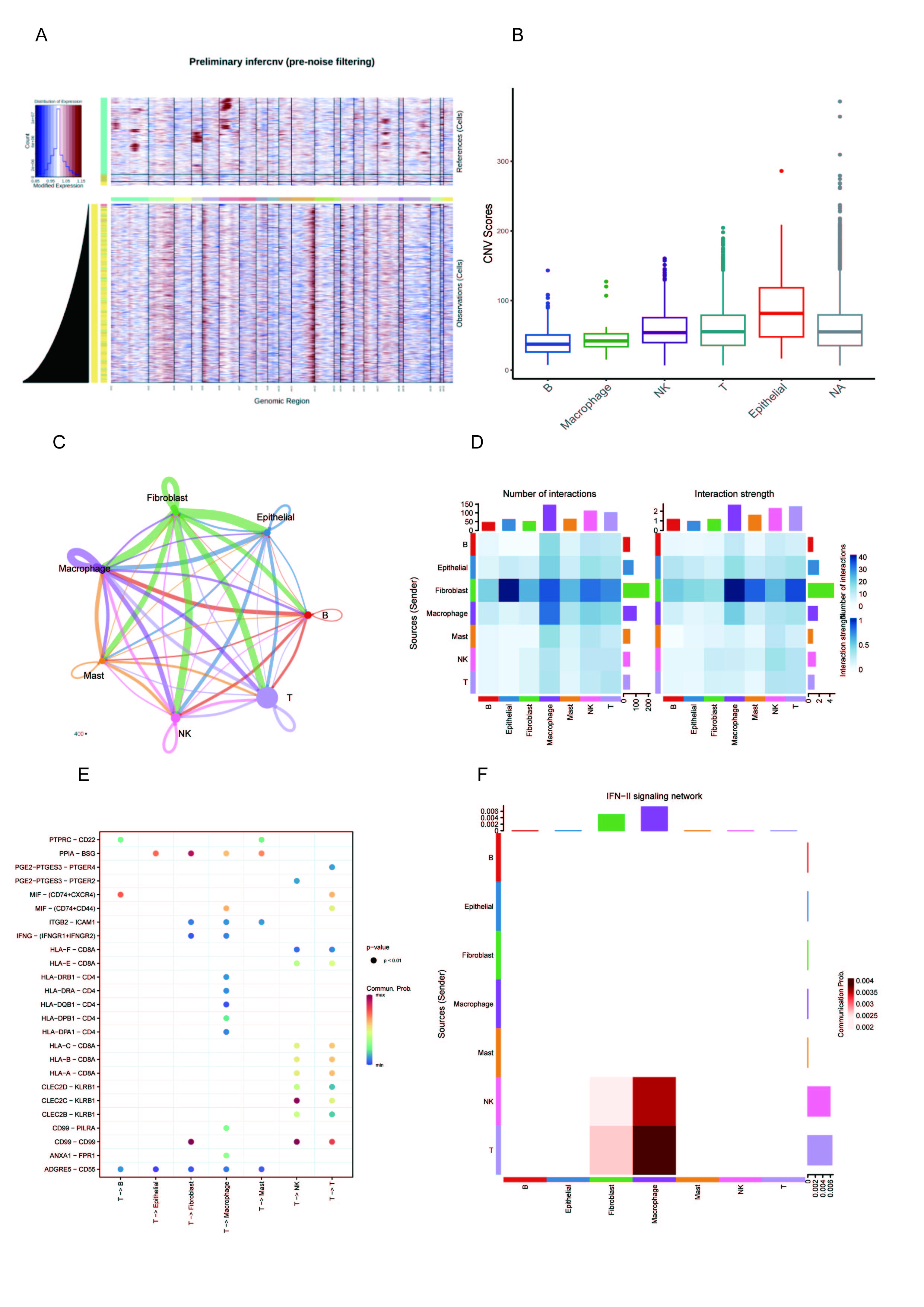

Supplement: Supplementary file 2 — Figure S2. | Analysis of malignant cells in CRC. (A) Dentifying large‐scale chromosomal copy number variations by InferCNV. (B) The box plots illustrate CNV scores, with epithelial cells exhibiting the highest scores, indicating their heightened susceptibility to transforming into malignant tumour cells. (C) The circle plot illustrates the communication network among immune cells, epithelial cells and fibroblasts in CRC. (D) The heatmap displays the number and intensity of interactions between immune cells, epithelial cells and fibroblasts. (E) Display and calculate the intensity of ligand–receptor interactions, and demonstrate the specific communication between cells through specific ligand–receptor interactions. (F) Heatmap shows the cell interaction strength of IFN‐II signalling network. [file JCMM-28-e70101-s005.jpg]

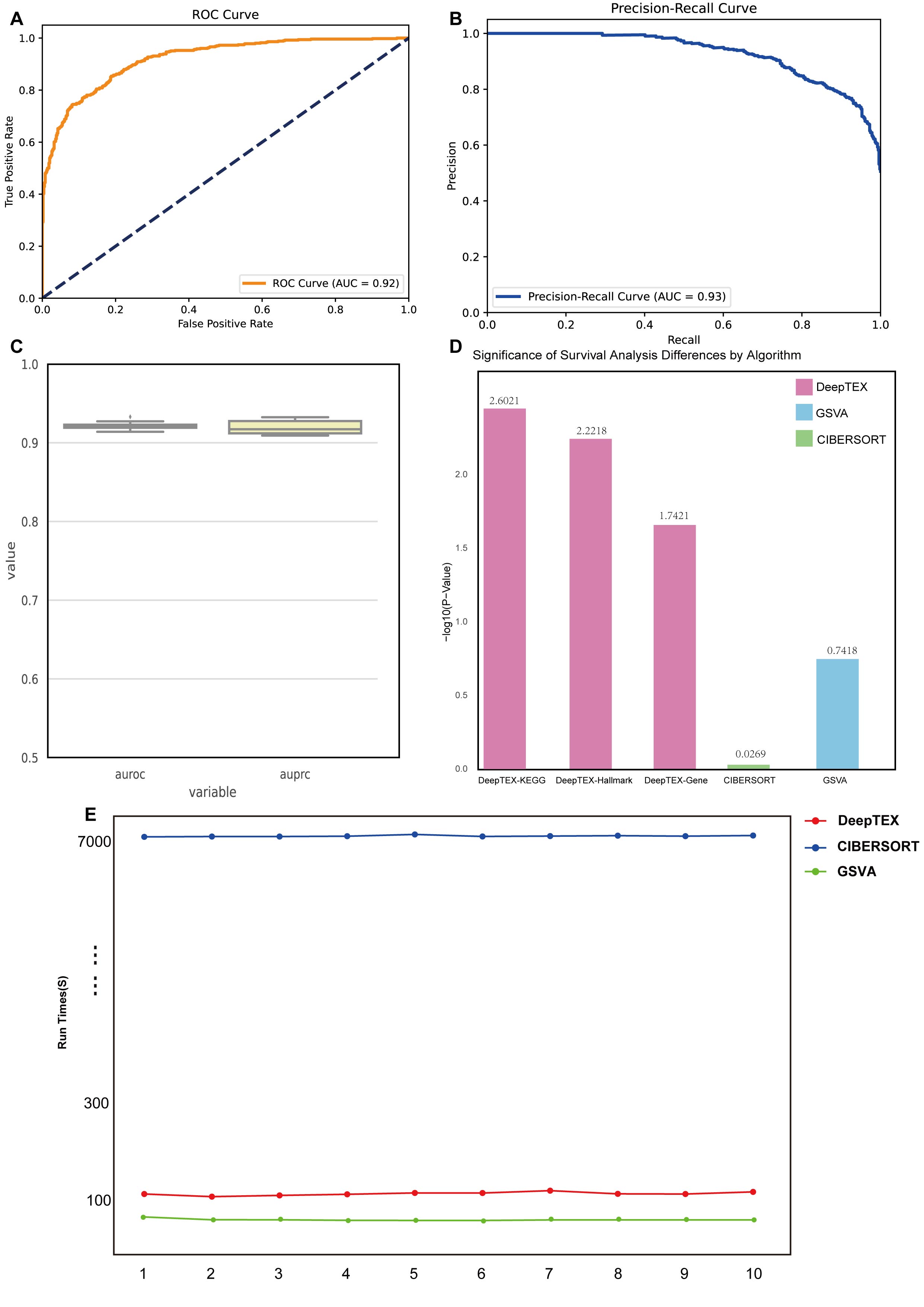

Supplement: Supplementary file 3 — Figure S3. | Performance Evaluation of DeepTEX in T‐cell exhaustion identification. (A) The diagnostic efficacy of DeepTEX was assessed through ROC curve analysis, which yielded an AUC of 0.92. (B) The precision‐recall curve analysis demonstrated an AUC of 0.93 (C) The boxplot demonstrates that DeepTEX achieves AUROC and AUPRC values greater than 0.9 for identifying exhausted T cells. (D) The histogram illustrates the significance of survival differences observed in the algorithm survival analysis. For predicting T‐cell exhaustion using KEGG, HALLMARK and GENE signatures, DeepTEX achieved (−log10) p_value of 2.6021, 2.2218 and 1.7421, respectively, compared to 0.0269 for CIBERSORT and 0.7418 for GSVA. (E) Line chart shows the performance of DeepTEX in significance of survival analysis differences and run time compared with other models. [file JCMM-28-e70101-s003.jpg]

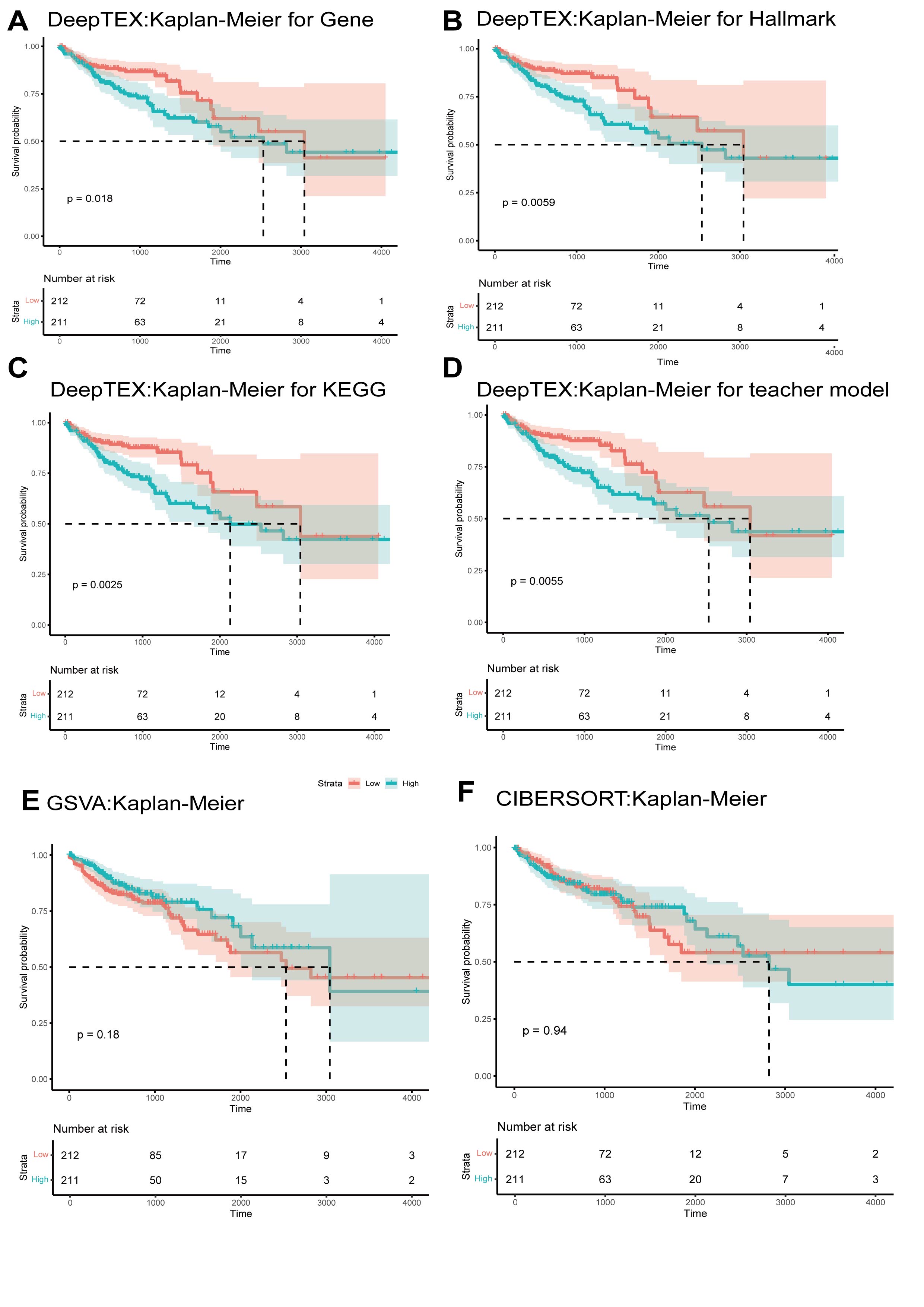

Supplement: Supplementary file 4 — Figure S4. | Survival Analysis Validation for DeepTEX. (A–D) DeepTEX performs survival analysis on CRC patients from the perspectives of Gene, Hallmark, KEGG and Teacher model. (E, F) The Kaplan–Meier curves for overall survival rates of T‐cell exhaustion in CRC using GSVA and CIBERSORT methods. ***p < 0.001, **p < 0.01, *p < 0.05. [file JCMM-28-e70101-s006.jpg]

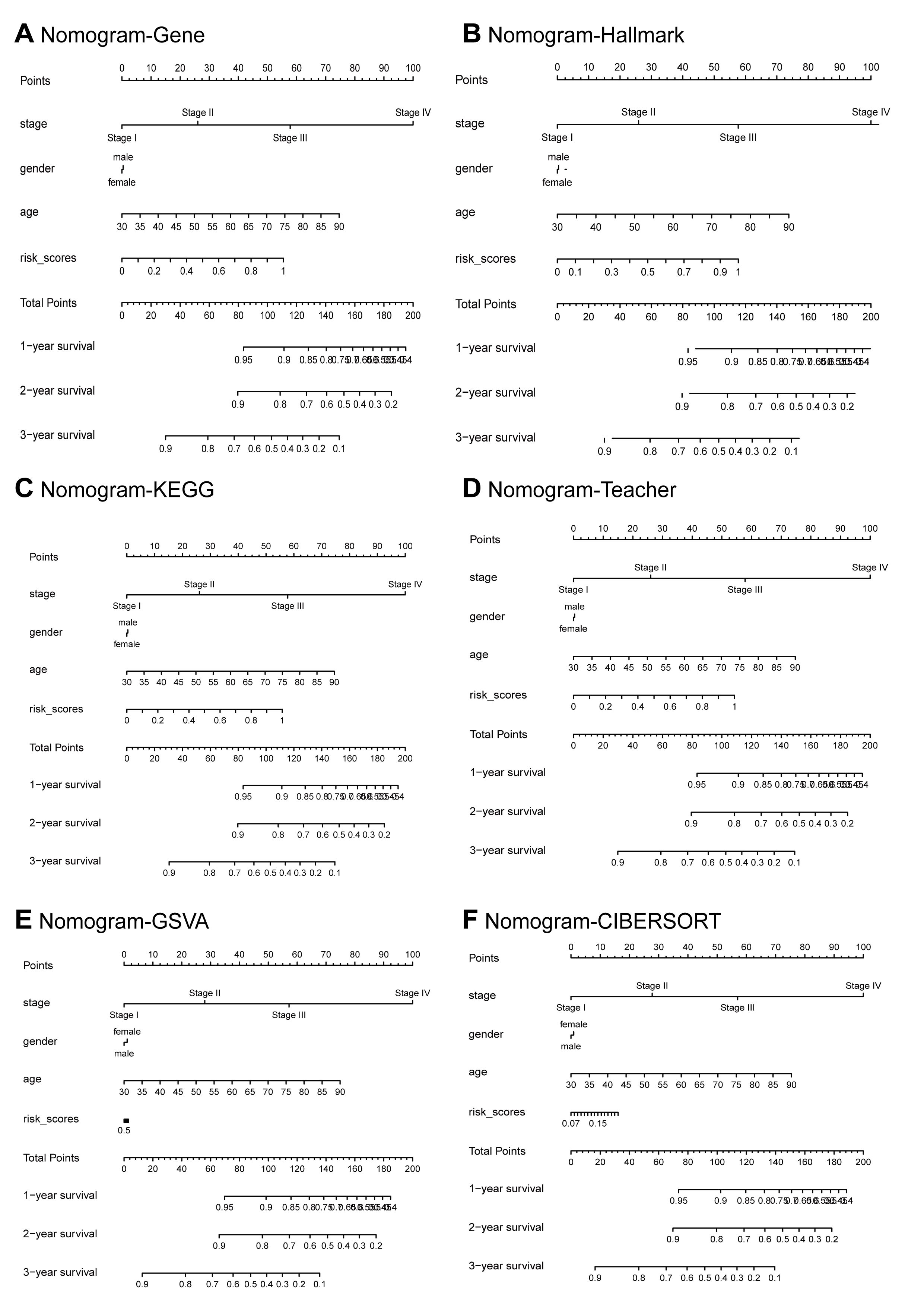

Supplement: Supplementary file 5 — Figure S5. | Risk Scores for T‐cell exhaustion in CRC Using DeepTEX, GSVA and CIBERSORT Nomograms. (A–D) DeepTEX performs risk scores on CRC patients from the perspectives of Gene, Hallmark, KEGG and Teacher model. (E, F) The nomograms for risk scores of T‐cell exhaustion in CRC using GSVA and CIBERSORT methods. [file JCMM-28-e70101-s008.jpg]

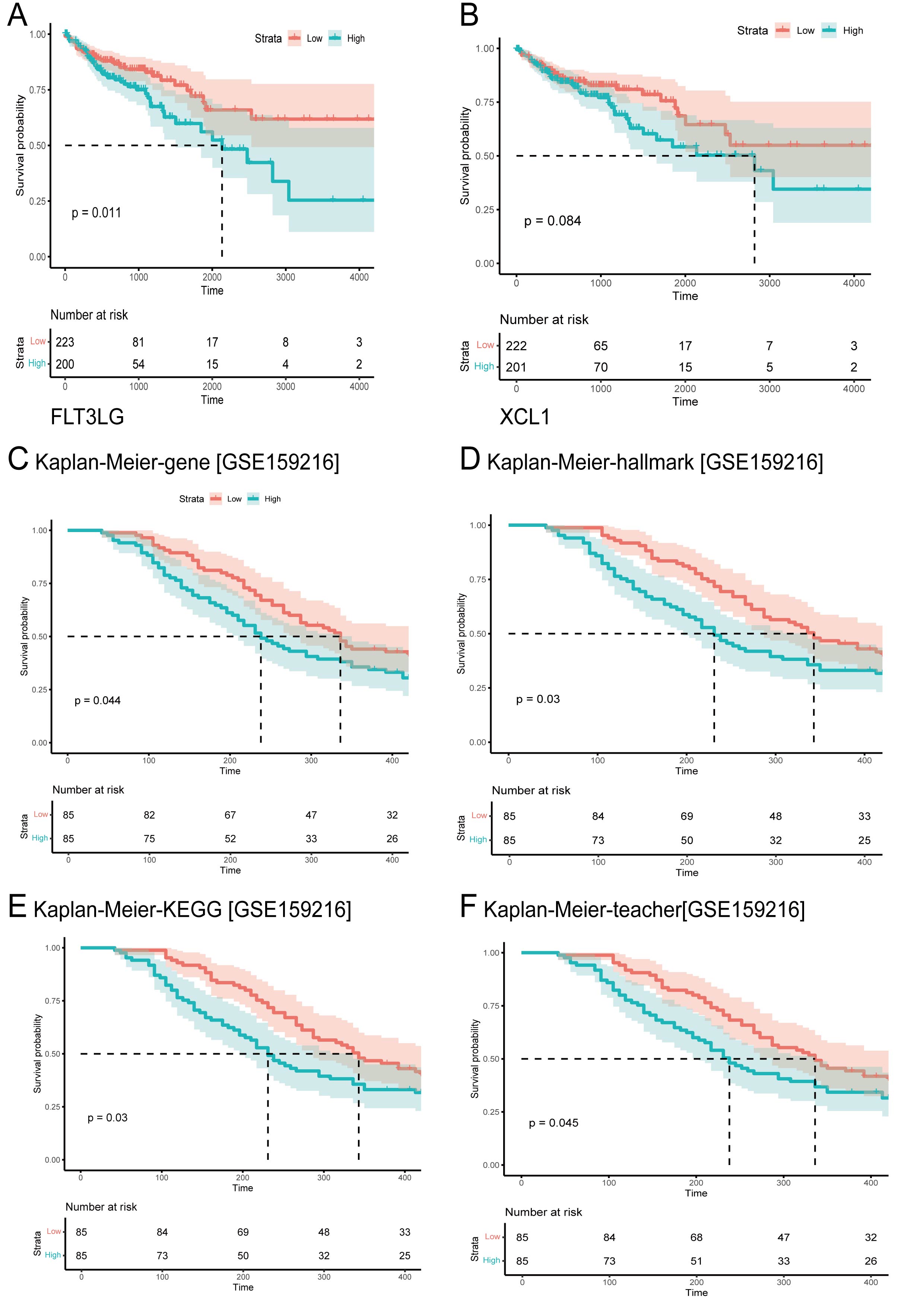

Supplement: Supplementary file 6 — Figure S6. | Validation of the DeepTEX (A, B) the prognostic significance of FLT3LG and XCL1. (C–F) GSE159216 CRC dataset to comprehensively evaluate the capability of the DeepTEX model in predicting T‐cell exhaustion related genes in CRC, across four distinct aspects: Gene, Hallmark, KEGG and Teacher model. ***p < 0.001, **p < 0.01, *p < 0.05. [file JCMM-28-e70101-s002.jpg]
