# Supplementary material for: Cross‐modal integration of bulk RNA‐seq and single‐cell RNA sequencing data to reveal T‐cell exhaustion in colorectal cancer
Source: J Cell Mol Med. 2024 Sep 29;28(18):e70101. doi: 10.1111/jcmm.70101 (PMC11439987; doi:10.1111/jcmm.70101)
Supplement: Supplementary file 8 — Data S1: [file JCMM-28-e70101-s001.docx]

**Supplementary Material**

**Supplementary Methods**

**1.1 Data Source and preprocessing**

We collected scRNA-seq sequencing data of CRC patients from public databases, among which the dataset for GSE178318 was downloaded from the Gene Expression Omnibus (GEO) database (https://www.ncbi.nlm.nih.gov/geo/), including sequencing sample data from primary, liver metastasis, and blood-derived CRC sources. In order to verify the accuracy of DeepTEX model in predicting T cell exhaustion in CRC, we also used GSE159216 data to verify the model. Extensive CRC transcriptome data and clinical information from The Cancer Genome Atlas (TCGA) database were downloaded from the GDC database (https://portal.gdc.cancer.gov/). For a relatively accurate assessment of factors associated with the survival of CRC patients, we screened the sample information, removing samples lacking survival information, and ultimately selected 424 patients for further analysis (Table S1-2).We used the "Read10X" and "CreateSeuratObject" functions of the "Seurat" package in R software (version 4.2.2) (https://www.r-project.org/),(https://mirrors.tuna.tsinghua.edu.cn/CRAN/src/base/R-4/R-4.2.2.tar.gz) to convert CRC samples into "Seurat" objects, using the "subset" function to filter for cells containing 400–4000 features and less than 10,000 counts. Due to the common manifestation of excessive mitochondrial contamination in damaged and dead cells, it was necessary to filter out cells with high mitochondrial gene expression. To remove cells with excessively high mitochondrial gene expression ratios without significantly losing sample cell information, we excluded cells where the percentage of mitochondrial genes exceeded 20%. Through quality control, we finally obtained 6,518 primary CRC cells in the scRNA-seq sample. 10,145 cells with liver metastasis from CRC; And 2,992 cells were transferred from peripheral blood. Extensive CRC transcriptome data and clinical information from The Cancer Genome Atlas (TCGA) were downloaded from the GDC database (<https://portal.gdc.cancer.gov/>). For a relatively accurate assessment of factors associated with the survival of CRC patients, we screened the sample information, removing samples lacking survival information, and ultimately selected 424 patients for further analysis. The "NormalizeData" function of the "Seurat" package is used for data normalization, employing "FindVariableFeatures" to select 2000 highly variable genes (HVGs) followed by normalization of the data using "ScaleData". Subsequent steps include Principal Component Analysis (PCA) and visualization of the data through Uniform Manifold Approximation and Projection (UMAP) method. The "FindClusters" method clusters the cells, after which a cell atlas is constructed using known cell type markers. "FindAllMarkers" is used to identify differentially expressed genes (DEGs).

1.2 Multi-Omics analysis

1.2.1 Bulk-RNA-seq DEGs and PPI

Samples were categorized into "Normal" and "Tumor" by combining CRC transcriptome data and clinical information. Differential expression analysis was conducted using the "limma" R package. Genes with a fold change of at least 2 and a Benjamini-Hochberg (BH) adjusted p-value less than 0.05 were selected as significantly changed genes. Subsequently, we used the "STRINGdb" R package to construct a PPI network using genes from the intersection of DEGs from Bulk RNA-seq and marker genes for exhausted T cells. By calculating the degree of each protein node, we selected the top 50 genes and the intersection genes as feature genes for downstream analysis.

**1.2.2 GSVA, DEGs and CNV analysis**

The "GSVA" package was used to convert gene expression profiles into pathway activity scores, with pathway data sourced from MSigDB (<https://www.gsea-msigdb.org/gsea/msigdb>). Visualization was performed using the "ComplexHeatmap" package. Pathway activity scores were divided into two groups, primary and metastatic CRC, for differential expression analysis. In the differential expression analysis, pathways with an adjusted p-value < 0.05 were considered to have statistical significance. To identify chromosomal copy number variations, InferCNV (<https://github.com/broadinstitute/inferCNV>) was employed to explore pathway activity in individual cells within scRNA-seq datasets of primary tumors compared to normal scRNA-seq datasets (Figure S2A-B).

**1.2.3 Cell communication analysis**

The R package "CellChat" enables cell interaction analysis in scRNA-seq data to uncover communication networks between cells. It is capable of identifying interacting cell groups at the single-cell level and aids in understanding the signaling among them. To explore the potential communication between T cells and other cell types in CRC and liver metastases, the R package "CellChat" was used to analyze ligand-receptor interactions among different cell types.

1.2.4 T cell SCNICE and trajectory analysis

We used SCENIC to infer the core transcription factors in T cell clusters. Monocle2 was employed for trajectory analysis of T cell clusters. Subsequently, we conducted differential gene expression analysis using the "differentialGeneTest" function to identify significant genes (BH corrected p < 0.01). High-variance genes were selected using the "setOrderingFilter" function. The "reduceDimension" function was then used for dimensionality reduction, followed by cell ordering using the "orderCells" function. Finally, trajectory construction and visualization were performed using "plot_cell_trajectory".

1.2.5 Analysis of T cell exhaustion stage

T cell clusters were subjected to dimensionality reduction, standardization, clustering, and visualization. We manually curated literature to collect marker genes related to T cell exhaustion, and using these markers, we categorized T cells into five main stages: non-exhausted stage, TEX int1, TEX int2, TEX prog, and TEX term. Using the "GSVA" software, we visualized the activity scores of TNF, IL2, and IFNG pathways across different exhaustion stages in CRC samples from various locations.

Supplementary Results

2.1 Performance Evaluation of DeepTEX in T Cell Exhaustion Identification

We have used newly generated pseudo-bulk data and a set of newly added bulk data to validate the effectiveness and generalization capability of our model. Specifically, for the pseudo-bulk data at different stages of T cell exhaustion, we employed performance metrics such as the Area Under the ROC Curve (AUROC) and the Average Precision of the Precision-Recall Curve (AUPRC) for the validation of the domain adaptation model (Figure S3). For the newly added bulk dataset, we used a pre-trained domain adaptation model to predict T cell exhaustion scores. In this investigation, we employed DeepTEX, a state-of-the-art deep learning framework, to discern the state of T cell exhaustion. The diagnostic efficacy of DeepTEX was assessed through ROC curve analysis, which yielded an AUC of 0.92 (Figure S3A), signifying a robust capability for identifying exhausted T cells. Furthermore, the precision-recall curve analysis demonstrated an AUC of 0.93 (Figure S3B), confirming the high precision and recall performance of DeepTEX in the classification of T cell exhaustion. We separately validated that both the domain adaptation model and the knowledge distillation model can effectively distinguish patient survival, demonstrating the excellent generalization ability of DeepTEX (Figure S3C).

**2.2 Compared with CIBRTSORT and GSVA.**

DeepTEX performs survival analysis on CRC patients from the perspectives of gene, hallmark, KEGG, and teacher model. Kaplan-Meier survival curves demonstrate that DeepTEX has a significant advantage over single-modal methods in analyzing T cell exhaustion in CRC. By comparing the prognostic survival of CRC patients with existing methods CIBRTSORT and GSVA, we found that DeepTEX exhibits higher significance in predicting the prognostic survival of CRC patients (Figure S4). It shows the performance of DeepTEX in significance of survival analysis differences and run time compared with other models (Figure S3E). To visually demonstrate the accuracy of DeepTEX in identifying T cell exhaustion, we compared the advantages of DeepTEX over GSVA and CIBERSORT in terms of accuracy in recognizing the degree of T cell exhaustion by using KEGG pathways, HALLMARK, and genes as inputs (Figure S4). When we input KEGG to predict the degree of T cell exhaustion, the (-log10)p_value for DeepTEX was 2.6021. while for CIBERSORT it was 0.0269, and for GSVA, it was 0.7418. When we input HALLMARK to predict the degree of T cell exhaustion, the (-log10)p_value for DeepTEX was 2.2218, while for CIBERSORT it was 0.0269, and for GSVA, it was 0.7418. When we input GENE to predict the degree of T cell exhaustion, the (-log10)p_value for DeepTEX was 1.7421, while for CIBERSORT it was 0.0269, and for GSVA, it was 0.7418 (Figure S3D). These results illustrate that DeepTEX significantly outperforms both GSVA and CIBERSORT in terms of statistical significance when predicting the degree of T cell exhaustion, as indicated by the higher (-log10)p_value scores for DeepTEX across all input types. This highlights the superior accuracy of DeepTEX in identifying T cell exhaustion compared to the other methods.

**2.3 Validation of the DeepTEX**

We selected the GSE159216 CRC dataset to comprehensively evaluate the capability of the DeepTEX model in predicting T cell exhaustion related genes in CRC, across four distinct aspects: Gene, Hallmark, KEGG, and Teacher model. Analyzing the prediction results with Kaplan-Meier survival curves, we found that the DeepTEX model yielded statistically significant p-values of 0.044, 0.03, 0.03, and 0.045 for the Gene, Hallmark, KEGG, and Teacher model, respectively (Figure S6 C-F). These findings underscore the robust predictive power of the DeepTEX model across different biological contexts and highlight its potential in elucidating the molecular mechanisms underlying T cell exhaustion in CRC. Furthermore, we conducted a comparative analysis of DeepTEX with two established methods, GSVA and CIBERSORT, in terms of their ability to discern the extent of T cell exhaustion. Notably, GSVA yielded a p-value of 0.18 (Figure S4E), indicating a less significant association, while CIBERSORT exhibited a p-value of 0.94 (Figure S4F), suggesting a notably weaker correlation. In contrast, DeepTEX, leveraging its comprehensive framework that encompasses Gene expression, Hallmark signatures, KEGG pathways, and Teacher model predictions, demonstrated statistically significant associations with p-values consistently below 0.05 across all four dimensions. This comprehensive approach underscores the potential of DeepTEX in accurately identifying markers of T cell exhaustion. The DeepTEX model outperforms GSVA and CIBERSORT in assessing the risk of T cell exhaustion in CRC by leveraging knowledge distillation and domain adaptation alignment methods (Figure S5). A comparative analysis of risk scores derived from the DeepTEX, GSVA, and CIBERSORT nomograms reveals that DeepTEX exhibits significant advantages in scoring T cell exhaustion risk in CRC across four dimensions: Gene, Hallmark, KEGG, and Teacher model. DeepTEX not only accurately stratifies patients based on their TEX status but also shows strong correlation with clinical outcomes, underscoring its potential in guiding personalized immunotherapy strategies and prognostic assessment. In summary, our findings highlight the superiority of DeepTEX as a robust tool for TEX identification in CRC, advancing the field towards more precise and effective cancer immunotherapies.

Using the DeepTEX model, we predicted key genes associated with cancer T cell exhaustion. Of note, we analyzed the prognostic significance of FLT3LG and XCL1, both of which were identified as potential biomarkers. Through Kaplan-Meier survival curve analysis, we observed statistically significant correlations with p-values of 0.011 and 0.084, respectively, indicating that DeepTEX can aid in predicting the potential roles of these marker genes in regulating T cell exhaustion and disease progression (Figure S6 A-B).

**2.4 In-depth analysis of T cell exhaustion mechanisms in primary CRC and liver metastases**

Eleven clusters were manually annotated as seven cell types, including B cells, epithelial cells, fibroblasts, macrophages, mast cells, NK cells, and T cells. From the annotation results, a significant enrichment of T cells was observed, indicating a higher degree of immune infiltration in patients with CRC. To further explore the regulatory mechanisms of T cell exhaustion in primary CRC and liver metastases, we selected T cells for sub-class annotation and analysis, visualizing T cell clustering using UMAP. Through integrated human CRC data, we identified the representative marker genes of the main cell clusters associated with T cell exhaustion**.** We manually annotated fourteen cell clusters into six cell types, including Naive T, TEX int1,TEX int2, TEX prog, TEX term, and Th17 [1]. Naive T Cells were annotated through the expression of marker genes SELL, IL7R, LEF1, CCR1, TCF7 [2]. During the pre-exhausted phase of CD8+ T cells, including TEX int1, TEX int2, and TEX prog, genes such as PDCD1, GZMH, CXCL13, GZMA, PRF1, GZMB, IFNG, and IFI16 are significantly expressed. TNFRSF9 and TIGIT show significant expression in TEX term. CRTAM, XCL2, TGFB1, STAT4, and GZMK are significantly expressed in Th17 cells (Figure 3) [3].

**Supplementary References**

1. Zeng Z, Wei F, Ren X. Exhausted T cells and epigenetic status. Cancer Biol Med 2020; 17:923–936

2. Kuang T, Zhang L, Chai D, et al. Construction of a T-cell exhaustion-related gene signature for predicting prognosis and immune response in hepatocellular carcinoma. Aging (Albany NY) 2023; 15:5751–5774

3. Tosolini M, Kirilovsky A, Mlecnik B, et al. Clinical impact of different classes of infiltrating T cytotoxic and helper cells (Th1, th2, treg, th17) in patients with colorectal cancer. Cancer Res 2011; 71:1263–1271

**Supplementary Figure legends**

**Figure S1 |** **Liver metastasis in CRC is associated with a higher degree of T cell exhaustion. A** Box plots show the number of cells at different stages of T cell exhaustion in primary CRC, metastatic cancer, and PBMCs. Liver metastasis exhibits a higher degree of terminal exhaustion compared to primary CRC. **B** Expression of IFNG, PDCD1, TNFRSF9 and TIGIT at various stages of T cell exhaustion. **C** Calculation of highly variable genes between primary CRC and liver metastasis, blood metastasis. **D** Monocle single-cell pseudotime trajectory analysis for primary CRC and liver metastasis, blood metastasis. **E** GSVA for primary CRC and liver metastasis.

**Figure S2 | Analysis of malignant cells in CRC. A** Dentifying large-scale chromosomal copy number variations by InferCNV. **B** The box plots illustrate CNV scores, with epithelial cells exhibiting the highest scores, indicating their heightened susceptibility to transforming into malignant tumor cells. **C** The circle plot illustrates the communication network among immune cells, epithelial cells, and fibroblasts in CRC. **D** The heatmap displays the number and intensity of interactions between immune cells, epithelial cells, and fibroblasts. **E** Display and calculate the intensity of ligand-receptor interactions, and demonstrate the specific communication between cells through specific ligand-receptor interactions. **F** Heatmap shows the cell interaction strength of IFN-Ⅱ sinaling network.

**Figure S3 |** **Performance Evaluation of DeepTEX in T Cell Exhaustion Identification. A** The diagnostic efficacy of DeepTEX was assessed through ROC curve analysis, which yielded an AUC of 0.92. **B** The precision-recall curve analysis demonstrated an AUC of 0.93 **C** The boxplot demonstrates that DeepTEX achieves AUROC and AUPRC values greater than 0.9 for identifying exhausted T cells. **D** The histogram illustrates the significance of survival differences observed in the algorithm survival analysis. For predicting T cell exhaustion using KEGG, HALLMARK, and GENE signatures, DeepTEX achieved (-log10)p_value of 2.6021, 2.2218, and 1.7421, respectively, compared to 0.0269 for CIBERSORT and 0.7418 for GSVA. **E** Line chart shows the performance of DeepTEX in significance of survival analysis differences and run time compared with other models.

**Figure S4 |** **Survival Analysis Validation for DeepTEX. A-D** DeepTEX performs survival analysis on CRC patients from the perspectives of Gene, Hallmark, KEGG, and Teacher model. **E-F** The Kaplan-Meier curves for overall survival rates of T cell exhaustion in CRC using GSVA and CIBERSORT methods. ***p < 0.001, **p < 0.01, *p < 0.05.

**Figure S5 | Risk Scores for T Cell Exhaustion in CRC Using DeepTEX, GSVA, and CIBERSORT Nomograms. A-D** DeepTEX performs risk scores on CRC patients from the perspectives of Gene, Hallmark, KEGG, and Teacher model. **E-F** The Nomograms for risk scores of T cell exhaustion in CRC using GSVA and CIBERSORT methods.

**Figure S6 |** **Validation of the DeepTEX A-B** The prognostic significance of FLT3LG and XCL1. **C-F** GSE159216 CRC dataset to comprehensively evaluate the capability of the DeepTEX model in predicting T cell exhaustion related genes in CRC, across four distinct aspects: Gene, Hallmark, KEGG, and Teacher model. ***p < 0.001, **p < 0.01, *p < 0.05.

**Supplementary Table Captions**

**Table S1. Summary statistics of all datasets enrolled in this work.**

**Table S2. TCGA_CRC Patients clinical information.**

**Table S3. HALLMARK-GSVA matrix.**

**Table S4. KEGG-GSVA matrix.**

**Table S5. Gene importance score**

**Table S6. HALLMARK importance score.**

**Table S7. KEGG importance score.**

**Table S8. Risk score (TCGA bulk data as model input).**

**Table S9. Risk score (HALLMARK-GSVA as model input).**

**Table S10. Risk score (KEGG-GSVA as model input).**

**Table S11. Deconvolution score of T cell exhaustion stage.**

**Table S12. Teacher network risk scores of DeepTEX.**

**Table S13. Data collection information comparison.**
